# Emancipating Sexuality: Breakthroughs into a Bulwark of Tradition

**DOI:** 10.1007/s11205-015-1137-9

**Published:** 2015-10-28

**Authors:** Amy C. Alexander, Ronald Inglehart, Christian Welzel

**Affiliations:** 1 The Quality of Government Institute (QoG), University of Gothenburg, P.O. Box 711, 40530 Gothenburg, Sweden; 2 Institute for Social Research (ISR), University of Michigan, Thompson Street, Ann Arbor, MI 48008 USA; 3 Laboratory for Comparative Social Research (LSCR), National Research University-Higher School of Economics, Moscow and St. Petersburg, Russia; 4 Center for the Study of Democracy (CSD), Leuphana University, Scharnhorststr. 1, 21335 Lüneburg, Germany

**Keywords:** Cultural change, Economic development, Emancipation, Emancipative values, Existential opportunities, Fertility norms, Freedom ladder, Life quality, Moral evolution, Religion, Secular values, Sexual liberation

## Abstract

This article presents evidence for a rising emancipatory spirit, across generations and around the world, in a life domain in which religion hitherto blocked emancipatory gains: sexual freedoms. We propose an explanation of rising emancipative values that integrates several approaches into a single idea—the utility ladder of freedoms. Specifically, we suggest that *objectively* improving living conditions—from rising life expectancies to broader education—transform the nature of life from a source of threats into a source of opportunities. As life begins to hold more promise for increasing population segments, societies climb the utility ladder of freedoms: practicing and respecting universal freedoms becomes increasingly vital to take advantage of rising life opportunities. This trend has begun to spill over into a life domain in which religious norms have until recently been able to resist emancipatory gains: sexual freedoms. We present (1) crossnational, (2) longitudinal, (3) generational and (4) multilevel evidence on an unprecedentedly broad basis in support of this theory.

**Electronic supplementary material:**

The online version of this article (doi:10.1007/s11205-015-1137-9) contains supplementary material, which is available to authorized users.

## Introduction

With the liberal revolutions of the Enlightenment era, human history has taken a sharp turn (Grayling 2007; Goldstone 2009). Ever since, struggles for emancipatory gains—from the abolition of slavery to the enforcement of human rights—have become a defining feature of modernity, and increasingly so (Markoff 1996; Tilly and Wood 2009; Pinker 2011). Of course, this does not mean that modernity is an uninterrupted chain of emancipatory triumphs. On the contrary, emancipatory struggles regularly meet the resistance of reactionary forces, including right-wing extremism, armed terrorism and religious fundamentalism (Armstrong 2001; Weinberg and Pedahzur 2004). Nevertheless, social movements, civil society actors and advocacy groups around the world continue to campaign for emancipatory gains, and they have been pushing the frontline of these gains to ever new fields (Clark 2009; Carter 2012).

More recently, the frontline has reached a domain in which religion and other conservative forces have been most successful in blocking emancipatory gains: sexual freedoms (Kafka 2005; Knudsen 2006; Frank et al. 2010).^1^ Indeed, legalization of abortion and same-sex marriage, together with laws against the discrimination of homosexuals, mark emancipatory breakthroughs at the societies’ reproductive grassroots: households, families and the sexual norms that shape their fabric (Sunder 2003; Alexander and Welzel 2011a; Asal et al. 2013). Here, emancipatory gains touch on a most fundamental domain of freedoms: the possession of our bodies and self-determination over our sexuality.

As this article will show, sexual freedoms represent the youngest domain in which we observe a rising emancipatory spirit. Societies marching towards sexual emancipation have made emancipatory gains in other areas, such as women’s suffrage, already earlier (Knudsen 2006; Paxton and Hughes 2007). And despite counter-motions by religious forces in Nigeria, Turkey, Russia and elsewhere, emancipative values are rising at an exceptionally steep slope in the domain of sexual freedoms.

Emancipatory gains in sexual freedoms are linked with the “second demographic transition”—a trend towards longer education, later marriage, alternative forms of cohabitation and lower fertility (Lesthaeghe 2010). This article examines the motivational force driving this trend: rising mass support for sexual self-determination (Twenge et al. 2015b).

Support for sexual freedoms belongs to a broader set of emancipative values, which emphasize freedom of choice and equality of opportunities. However, among the various components of these values, support for sexual freedoms has emerged more recently and most rapidly. At the same time, sexual freedoms remain an especially contested domain of emancipation because conservative forces, most notably religion, concentrate their resistance here (Frank et al. 2010; Hildebrandt 2014; Doebler 2015).

In *Freedom Rising*, Welzel formulates an “evolutionary theory of emancipation” to explain emerging emancipative values (Welzel 2013, 2014). But the author does not apply this theory to sexual freedoms in particular. Given this domain’s exceptional dynamic and contestation, this is a critical omission. Our examination fills this gap, testing the theory of emancipation specifically with respect to sexual freedoms.

The theory of emancipation takes as a starting point the rising life expectancies in many parts of the world (Welzel 2013: 4). As people’s lifetime horizons extend, education and other investments in human development with a long delay of gratification gain appeal—an appeal that is absent when families are pre-occupied with reproduction to secure the continuation of their lineage in the face of short life expectancies (Woodley 2012). But where time horizons expand, the nature of life changes from a source of constraints into a source of opportunities. As this happens, societies climb the *utility ladder of freedoms*: universal freedoms become increasingly vital for using the opportunities offered by a more prospective—and for that matter—promising life.

The theory of emancipation posits that evolution has endowed humans with the ability to recognize life opportunities because this ability is essential for success. Thus, an objective ascension of opportunities does not escape people’s awareness and, hence, induces an adaptive shift in *subjective* values—giving rise to emancipatory orientations that support universal freedoms. This utility-value link is key to human functioning because it keeps our lives in touch with reality.

Sexual freedoms are of particular significance in this context. Throughout history, sexual reproduction has been the life domain in which tradition proved most powerful in blocking emancipatory gains. Propped up by religion, traditional norms in practically every culture emphasize kinship ties, family size and high fertility under male control of female sexuality (Smuts 1995; Norris and Inglehart 2003; Blumberg 2004; Lesthaeghe 2010; Alexander and Welzel 2011b). Against the perennial inertia of these traditional family, fertility and sex norms, rising emancipative values in the domain of sexual freedoms signal an evolutionary breakthrough in the development of moral systems. As we will demonstrate, this moral evolution is induced by expanding opportunity endowments in the lives of increasing population segments.

For the first time, this article presents evidence for these propositions on a global cross-cultural basis.^2^ In so doing, our examination breaks new ground in three ways. First, we examine the impact of an unprecedentedly wide range of opportunity-endowing societal conditions. Second, we pay special attention to dynamic patterns and temporal order in the co-evolution of emancipative values and opportunity endowments. Third, we examine emancipative values with a distinct focus on sexual freedoms, and we do so on the widest cross-national and longitudinal basis ever used in the study of values, covering a period of almost 30 years among societies representing more than ninety percent of the world population.

For reasons of brevity, the term “emancipative values” refers *specifically* to the domain of sexual freedoms, here and throughout the remainder of this article—which is organized in four sections. Section one presents a framework to integrate disparate explanations of emancipative values and anticipates the key findings derived from this framework. Section two describes the data and methods used to demonstrate these findings. Section three illustrates them. The final section discusses the implications and limitations of the evidence.

## Theory: The Utility Ladder of Freedoms

The idea of a *utility ladder of freedoms* constitutes the pivotal principle of Welzel’s evolutionary theory of emancipation (Welzel 2013: 37–56, 2014: 48). The idea involves two premises:

1. *Variability in Freedoms’ Utility.* Guarantees of universal freedoms have varying utility for people in how to master their lives: the utility of such guarantees grows when people’s existential conditions embody more options for intentional action. Then, more guarantees are needed to protect people’s choices on how to use their options. With few options for intentional action, legal guarantees of choices are rather useless.
2. *The Utility*-*Value*-*Link.* Human life strategies are shaped by a utility-value link: people tend to value what is useful for mastering life under given circumstances. This utility-value link keeps human existence in touch with reality. Hence, if expanding life opportunities enhance the utility of guaranteed freedoms in an *objective* sense, *subjective* values adjust in the same direction, towards supporting freedoms.

Literatures from different disciplines support these premises. These literatures include: (a) works on the “democratic character” (Lasswell 1951) and its opposite, the “authoritarian personality” (Adorno et al. 1950; Rokeach 1960; Duckit and Bizumic 2013); (b) studies in experimental psychology on “preventive” versus “promotive” orientations and on weak versus strong “social dominance orientations” (Higgins et al. 1998; Higgins 2005; Sidanius et al. 1994, 2000); (c) research in sociobiology on the cultural consequences of disease stress (Thornhill et al. 2009; Murray et al. 2013); (d) investigations in political sociology on liberal orientations (Stouffer 1955; Sullivan et al. 1982; Brint 1984; Lamont 1987; Sullivan and Transue 1999) and its opposites—right-wing extremism and religious fundamentalism (Jackson et al. 2001; Scheve and Slaughter 2001; Givens 2005; Huddy et al. 2005; Wagner et al. 2006; Coenders et al. 2008); and (e) examinations in political culture on “survival-vs.-self-expression values” (Inglehart and Welzel 2005).

The central idea on which these literatures converge can be related to a contrast known in evolutionary psychology as “short” versus “long life histories” (Woodley 2012). “Short life histories” are characterized by high mortality and fertility and correlate with poverty, social immobility and lacking education. The constraints of these conditions leave people little choice about how to dedicate their time; they are permanently forced to meet their most pressing needs. Accordingly, people perceive life that way and see little value in freedoms that they could not use anyways. By contrast, “long life histories” are manifest in low mortality and fertility and associate with prosperity, social mobility and widespread education. These conditions leave people considerable choice about how to devote their time and shape their biographies. Consequently, people see life as a source of opportunities and value the freedoms that allow them to take advantage of these opportunities.

By far the largest part of human history is characterized by the “short life history” condition. Only since the Industrial Revolution have some limited segments of humanity began to overcome this dismal condition (Goldstone 2009). But since the last 40 years, most of the world population is a in transition towards “long life histories”: falling mortality and fertility, together with rising prosperity, education and social mobility expand the life opportunities of most populations around the globe (Estes 2010; Welzel 2013: 4). As life opportunities ascend on a mass scale, entire populations climb the utility ladder of freedoms and increasingly embrace emancipative values.

Three qualifications of this proposition are due. First, ascending life opportunities give rise to emancipative values in the domain of sexual freedoms *only insofar* as these opportunities promote secularization. The reason is religion’s intimate alliance with traditional reproduction norms. Decades of research confirm that religion—irrespective of the specific denomination—protects traditional reproduction norms and, thus, sustains a shield against emancipatory gains in this domain (Harkness 1972; Ruether 1974; Peek et al. 1991; Norris and Inglehart 2003; Burn and Busso 2005; Doebler 2015). Traditional reproduction norms are functional under the constraints of “short life histories”: when mortality, poverty and extreme inequality are prevalent, high female fertility under male control is instrumental for the continuation of family lineages (Blumberg 2004; Hudson et al. 2012). “Short life histories” provide also the condition under which religion is appealing: the promise of salvation from this-worldly misery makes it easier to accept dismal conditions (Becker 1973; Solomon et al. 1991; Jong et al. 2013). Hence, existential constraints create a natural alliance between religiosity and traditional sex norms (Inglehart and Norris 2011; Doebler 2015). Since ascending life opportunities diminish existential constraints, these opportunities erode the utility of traditional sex norms. Yet, emancipative values spread into the domain of reproductive freedoms only if religiosity declines under the felt utility loss of traditional sex norms. Secularization is, hence, an intermediate step from ascending life opportunities to sexual emancipation.

Second, emancipative values represent a socially *reciprocal* orientation: their emphasis on universal freedoms includes the freedoms of others, which is an attitude that one adopts more easily if others reciprocate the favor and value one’s own freedoms in return. To spread, these reciprocations need widely shared utilities, based on opportunity endowments that are *common* in a society. This proposition suggests that people’s emancipative values grow more on the basis of options they share with many others than on the basis of options they have on top of what most others have. In that sense, the utility ladder of freedoms is about socially shared utilities, not individually unique utilities.^3^

Third, ascending life opportunities are likely to give rise to emancipative values more strongly among younger generations because age increases the inertia of people’s formative values (Inglehart 1977; 2008). Hence, life opportunities measured over the time of a given birth cohort’s upbringing predict fairly well the cohort members’ emancipative values today.

These propositions inform five key findings that the evidence section will demonstrate:

1. Societies with greater life opportunities among the bulk of the population today exhibit higher *mean* levels of emancipative values.
2. Societies in which the life opportunities of the population improved more experienced greater *gains* in emancipative values.
3. Birth cohorts in societies whose existential conditions embodied greater life opportunities back in time exhibit stronger emancipative values today.
4. People’s emancipative values are strengthened more powerfully by life opportunities that are socially common than by opportunities that are individually unique.
5. On all these accounts, the advancement of emancipative values is conditional on corresponding advancements in secular values.^4^

## Data

### Sources and Sample

We measure emancipative values using the World Values Surveys (WVS 2010). At the time of this writing, the WVS completed five waves of representative national surveys from 1981 to 2008, with random samples of adult populations in countries around the globe (for documentation see: www.worldvaluessurvey.org). We report measurement details, data sources, descriptive statistics and provide links to replication data in an Online Appendix (OA), accessible at this journal’s website (http://www.springer.com/social+sciences/journal/11205). The OA also includes a response section where we clarify issues that came up in the review process. Data from the sixth wave of the WVS, conducted from 2011 till 2014, became available only after our analyses have been finalized. We have, however, tested if the trend towards rising emancipative values continues into wave six and the evidence is strongly confirmatory.

In support of our propositions, we present four distinct types of findings, including (1) cross-sectional, (2) longitudinal, (3) cohort-related and (4) multilevel evidence. From the cross-sectional perspective, we explain between-societal differences in emancipative values. For this analytical step, we use data from eighty-one to ninety-three societies worldwide. As the list of countries and their attribution to global cultural zones in Appendix-Table 1 (OA 1: 6) shows, these societies distribute quite evenly across all regions of the globe.

From a longitudinal point of view, we explain the direction and amount of change in emancipative values among those societies for which a considerable time series exists. This diminishes the sample to about fifty societies. But even this smaller sample includes the societies with the largest populations in each world region, as documented in OA 19 (p. 30). The smaller longitudinal sample shows similar variability in the key variables of interest as the larger cross-sectional sample. This is documented in Appendix-Table 9 (OA 19: 30).

From a generational perspective, we explain cohort differences in emancipative values within and between societies. Specifically, we demonstrate that the mean level of emancipative values in a given birth cohort is predicted by the respective country’s life opportunities at the time when the members of this cohort were growing up. The variables of interest are available for eighty-five societies of the cross-sectional sample, each being divided into six successive birth cohorts, as documented in OA 15 (p. 26). The unit of analysis is (85 × 6=) 510 country-cohorts.

From a multilevel perspective, we show how the emancipative values of some 130,000 respondents are shaped simultaneously by individual and societal characteristics. Data on the variables of interest are available for eighty-nine societies of the cross-sectional sample. Its composition is documented in Fig. 7.

### Variables

All variables measuring attitudes are taken from the WVS. Variables measuring structural characteristics of societies are taken from the Quality of Government Dataset (Quality of Government Institute 2010). Without exception, we standardize every variable into a range from minimum 0 to maximum 1, with fractions for intermediate positions. This makes regression coefficients comparable across variables originally measured in different coding schemes.

Our dependent variable, emancipative values in sexual freedoms, is a thirty-point index measuring a respondent’s acceptance of homosexuality, abortion and divorce, as documented in OA 1 (pp. 4–6). These are the only items that capture sexual freedoms throughout all consecutive rounds of the WVS. For a longitudinal analysis this is crucial.

The Cronbach’s alpha for the three items is .80 at the individual level and .85 at the society level. The three items represent a single dimension within and across global cultural zones (Appendix-Table 1, OA 1: 6). Using from each society (*N* = 93) the most recent survey, the mean score in emancipative values is .31. The standard deviation is .28.

**Fig. 1:**
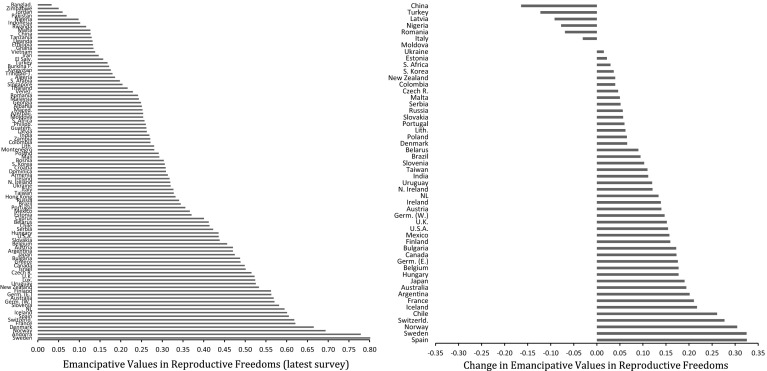
Levels and changes in emancipative values in sexual freedoms

To measure the prevalence of emancipative values throughout each society, we calculate the population average. As the left-hand diagram in Fig. 1 shows, the lowest scores in emancipative values are found in Bangladesh (.03), Zimbabwe (.05), Jordan (.06) and Nigeria (.09). The highest scores exist in Denmark (.67), Norway (.69), Andorra (.78) and Sweden (.80).

Emancipative values in sexual freedoms indicate an emancipatory worldview in a broader sense. This conclusion is corroborated by the fact that support for sexual freedoms represents one of four components in Welzel’s (2013) broader measure of emancipative values. The other components include an emphasis on (a) people’s voice, (b) personal autonomy and (c) gender equality and are each measured by three items, as shown in OA 2 (pp. 7–9). At the individual level, emancipative values in sexual freedoms correlate with emancipative values in these other fields significantly and positively at *r* = .23 (voice), .25 (autonomy) and .33 (equity). The society level correlations are .59, .60 and .73, in the respective order. Nevertheless, support for sexual freedoms sticks out from emancipative values in other domains: it represents an internally more coherent orientation and associates more closely with theoretically expected correlates of an emancipatory worldview.^5^ There is also evidence that support for sexual freedoms represents the frontline where emancipatory gains are both most recent and most contested. This can be seen from the fact that average support for sexual freedoms (.31) is lower than support for people’s voice (.36), personal autonomy (.44) and gender equality (.56). And support for sexual freedoms is least consensual: the variance coefficient is .90, compared to .75 for support for people’s voice, .70 for personal autonomy and .50 for gender equality.

These patterns qualify support for sexual freedoms as an orientation that is meaningfully related to other indications of an emancipatory worldview but at the same time distinct enough to justify a focus on precisely this field of emancipative values.

The right-hand diagram in Fig. 1 documents changes in emancipative values in the field of sexual freedoms, from the earliest to the latest survey for the fifty societies of our longitudinal sample.^6^ The average time distance covered is 17 years (for most societies, the earliest time point is around 1990 and the latest around 2005). The average change score over all fifty societies is +.10. Societies with positive changes above .05 scale points outnumber those with negative changes of that scope by 37 to 5. If we focus on the eleven postindustrial societies (footnoted in Fig. 5) that participated in both the first round of the WVS (1981) and the last one (2008), the mean change score over these 27 years is +.20. Across the same societies and time span, similar changes exist for the other components of emancipative values, albeit on a narrower scope: +.10 for gender equality, +.12 for people’s voice and +.18 for personal autonomy (Welzel 2013: Online Appendix, pp. 47–48).^7^

To explain emancipative values we champion opportunity endowments of an existential nature. Such endowments enhance people’s options for self-development. Following the “life history approach,” we assume that populations benefit from richer opportunities for self-development when (a) life expectancies are longer, (b) fertilities are lower, (c) access to education is broader, (d) per capita incomes are more equally distributed and (e) higher on average. To measure these life opportunities, we use a population’s (a) average life expectancy at birth, (b) its inverse fertility rate, (c) tertiary enrollment ratio and mean years of schooling, (d) the inverse of the Gini coefficient for income inequality, and (e) the per capita Gross Domestic Product (GDP) in purchasing power parities, all taken from the time at which emancipative values are measured (OA 6: 15–16). These indicators are equivalent to those combined by Woodley (2012) to measure the opportunity endowments of “long life histories.”

All life opportunity measures converge in a single dimension and the Cronbach’s alpha of the six indicators is .85. Thus, we summarize these measures into an encompassing *index of life opportunities* as detailed in OA 6. To straighten out a curved distribution, we square the original index.^8^

Apart from life opportunities, another source of permissive conditions comprises a society’s institutional qualities—qualities that make people’s lives safer and their options more certain and predictable.^9^ We label these qualities “bureaucratic integrity,” “law and order,” “civil supremacy” and “administrative accountability.” Data on these variables originate in the International Country Risk Guide and summarize expert judgments on safety from the risks of corruption (bureaucratic integrity), disorder (law and order), military takeovers (civil supremacy) and state chicanery (administrative accountability). The Risk Guide’s summary indicator of safety from all these risks (originally named “political risk”), is labeled *institutional functioning index* here (see OA 9: 19).^10^

Apart from institutional functioning, democracy is another source of permissive conditions because it entitles people to voice their policy preferences and make them count. When it comes to lasting entitlements, *enduring* democracy is important. To measure enduring democracy we use Gerring et al.’s (2005) “democracy stock index” (OA 10: 20–21). In addition, we use Welzel’s (2013: 253–260) *civic entitlements index* (OA 5: 14).

Besides formal institutions, cultural norms are a source of more restrictive or permissive conditions. Gelfand et al. (2011) describe cultures as “tight” when their norms are rigid and as “loose” when these norms are permissive. Likewise, Suh et al. (1998) attribute restrictive tendencies to “collectivist” norms and permissive tendencies to “individualistic” norms. We use these authors’ measures of tightness-vs.-looseness and collectivism-vs.-individualism as documented in OA 10 (pp. 20–21). Another manifestation of cultural restrictions is traditional reproduction norms, which are evident in “consanguine” marriage patterns and “patrilocal” household structures (Blumberg 2004; Hudson et al. 2012). Consanguine marriages happen among distant relatives; patrilocal households are formed when couples live with the husband’s parents. To measure consanguinity, we use the data from Woodley and Bell (2012). To measure patrilocality we calculate from the latest WVS for each society the fraction of married men above the age of thirty living in their parents’ household (see OA 10: 20–21).

An inverse indicator of traditional family, fertility and sex norms and, thus, a measure of permissive cultural environments is the prevalence of secular values. To measure secular values we use questions from the WVS covering three aspects of religiosity: the importance attributed to religion, self-description as a religious person and frequency of service attendance. As documented in OA 3 (pp. 10–12), all three measures are highly correlated, so we average them and then inverse the average to obtain a measure of secular values—indicating a distance to religiosity. To measure the prevalence of secular values, we calculate the national mean. This is an important, yet more concise, component of Welzel's (2013: 63–66) broader measure of secular values.

At the society level, measures of values no longer indicate personal preferences. Instead, they turn into measures of a society’s cultural tendency, indicating to what extent personal values represent shared collective norms (Welzel 2013: 84).^11^

Yet another domain of permissive conditions is the absence of violence and armed conflict. We use three indicators. Internal peace is the inverse of Gibney et al.’s (2010) “political terror scale,” measuring the absence of state repression. External peace is the inverse of a society’s international conflict involvement, taken from Gleditsch et al. (2002). Encompassing peace is based on the “global peace index” by the Vision of Humanity (2010), as documented in OA 10 (pp. 20–21).

To some extent, every society is influenced by its international environment but this influence is arguably stronger for societies that are more involved in international exchange. For instance, global discourses that advocate emancipative values have a stronger impact on societies that are more exposed to such discourses through higher rates of international exchange. Thus, the prevalence of emancipative values in a society might reflect its degree of exchange with the international environment. Hence, we use indicators of the societies’ economic, social and political exchange, plus a summary indicator labeled *global exchange index*. The data were collected by Dreher et al. (2008), as documented in OA 7 (p. 17).

Culture zone theories suggest that societies do *not* indifferently pick up anything from their international environment but are more receptive of trends in societies which they perceive as alike based on cultural similarities (Inglehart and Welzel 2005). Hence, a society’s level of emancipative values might reflect the level of these values in societies belonging to the same culture zone. To test this idea we create a *cultural diffusion index* that assigns each society the average level of emancipative values of all other societies of the same culture zone, based on the culture zone classification by Welzel (2013: 23–24) (see OA 8: 18).

In examining the cohort pattern of value change, we go back to living conditions several decades ago when older cohorts were in their formative years. Since this temporal extension limits data availability, we focus on the two most distinctive types of permissive conditions, one of an existential and the other of an institutional nature: life opportunities and civic entitlements. Because of data restrictions, we rely on proxy measures of these variables, using Vanhanen’s (2003) combined literacy and urbanization estimates as a proxy for life opportunities and his index of democratization as a proxy for civic entitlements. OA 13–14 (pp. 24–25) provide a validation of these proxies.

To examine our theory’s micro-foundation, we introduce additional predictors of emancipative values at the individual level. These include a summary measure of the other three components of emancipative values (Welzel 2013: 57–104; OA 2: 7–9). The reason is obvious: since emancipative values in the field of sexual freedoms are supposed to grow on the basis of emancipative values in other fields, these other emancipative values should be a significant predictor of emancipative values in the field of sexual freedoms. The same should hold true for individual-level characteristics indicating a person’s opportunity endowments in an objective sense. A decent indicator of a person’s opportunity endowments is her level of education, which is measured on a nine-point index from incomplete primary-level to complete tertiary-level education. As routine demographic controls, we include biological sex and a respondent’s year of birth. These variables are documented in OA 18 (p. 32), with descriptive statistics shown in OA 22 (p. 39).

### Methods

We explain variation in emancipative values on four different bases of evidence. First, we explain contemporary differences in these values between societies, using our cross-sectional sample of eighty-four to ninety-three societies. In this analytical step, treatment variables are taken from the same year as the surveys used to measure emancipative values. We take the latest available measure for each society, which spans a period from 2000 to 2008.

Second, we explain the amount and direction of change in emancipative values among the fifty societies of our longitudinal sample, based on simultaneous change in those treatment variables that were significant in the cross-sectional analyses.

Third, we explain cohort differences in emancipative values within and between societies by the living conditions that our 510 country-cohorts experienced during the decade in which they were growing up, using temporally ordered panel regressions.

Fourth, we apply multilevel models to examine how individual and societal characteristics simultaneously shape the emancipative values of some 130,000 respondents from eighty-nine societies. Specifically, we demonstrate that opportunities which people have in common with most others in their society strengthen their emancipative values more pronouncedly than do opportunities which people have on top of what most others have.

## Evidence

### Cross-Sectional Evidence

We expect that permissive conditions in their various manifestations explain fairly well how prevalent emancipative values are in given societies. Indeed, the correlations in Appendix-Table 4 (OA 11: 22) show that each of the twenty-eight indications of permissive societal conditions correlates positively and significantly with the prevalence of emancipative values. Apart from cultural diffusion, three manifestations of permissive conditions show a particularly strong link with emancipative values: life opportunities, civic entitlements and secular values.

**Fig. 2:**
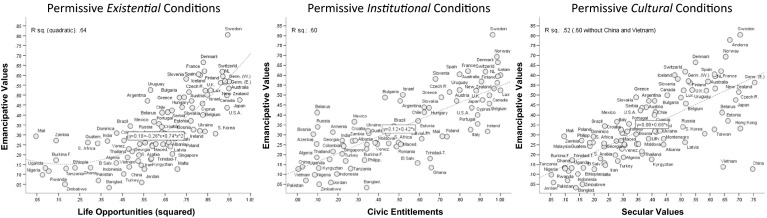
Links between emancipative values with regard to sexual freedoms and three manifestations of permissive societal conditions

Figure 2 visualizes these correlations and shows a similar grouping pattern throughout all three manifestations of permissive conditions: societies in Sub-Saharan Africa, South Asia and the Middle East beset the lower left-end of the distribution; Western societies led by Scandinavia are at the upper right end; societies from Latin America and the ex-communist world are found in between; societies from East Asia scatter over the entire space, with representatives at both the lower-left (e.g., Vietnam) and the upper-right end (e.g., Japan).

This pattern is familiar from countless studies of social development and few scholars would be surprised to rediscover it with whatever *objective* social indicator one uses (Delhey and Newton 2005; Estes 2010). However, we rediscover the same pattern with *subjective* indicators taken from mass survey data. This is noteworthy given the widespread suspicion that survey measures are not as reliable as objective social indicators (e.g., Stegmueller 2011). Disconfirming this suspicion, the strong link of emancipative values to life opportunities and civic entitlements reveals that aggregations of subjective values closely reflect objective societal conditions. In that very sense, these measures are real, providing powerful evidence that moral systems are in close touch with objective realities.

Looking at the relationship between secular values and emancipative values in the right-hand diagram of Fig. 2, China and Vietnam are outliers: they combine strong secular values with *weak* emancipative values. Other societies in East Asia—including Hong Kong, Japan, South Korea and Taiwan—also show weaker emancipative values than their strong secular values suggest. Arguably, this pattern reflects the role of Confucianism in East Asia. In history, religion was not a formative force of Confucian culture. Thus, religiosity is not the chief preservative of traditional reproduction norms in this particular culture. For this reason, strong secular values in East Asia are—in contrast to everywhere else—*not* an indicator of an emancipatory culture.

Permissive societal conditions are collinear across the different domains in which they materialize. This defies a “kitchen sink” approach that includes many conceptually related predictors at once in a multivariate regression to explain emancipative values. Instead, we select from each domain of permissive conditions only one indicator, namely that with the strongest link to emancipative values. Thus, we select life opportunities from the existential domain, civic entitlements from the institutional domain, secular values from the moral domain, and cultural diffusion from the domain of outside influences. In so doing, we combine breadth with parsimony, covering a broad range of distinct domains while selecting only the strongest indicator from each.

Table 1 shows the results of regressions that explain the prevalence of emancipative values by the selected indicators of permissive societal conditions. In the first model, life opportunities, civic entitlements and cultural diffusion all show an independent and significantly positive effect on emancipative values. Among these three conditions, cultural diffusion associates with a steeper increase in emancipative values than do life opportunities. But the larger T-ratio of the coefficient for life opportunities indicates that their explanatory power over emancipative values is larger than that of cultural diffusion.

**Table 1:** Cross-sectional explanation of societal-level emancipative values with regard to sexual freedoms

Predictors (at time of latest survey)	Dependent variable: emancipative values with regard to sexual freedoms (latest survey, 2000–2008)		
	Model 1	Model 2	Model 3
Constant	−.00 (−.30)^†^	−.00 (−1.70)^†^	.01 (.47)^†^
Life opportunities (sq.)	.21 (2.98)***	.05 (.65)^†^	
Civic entitlements	.15 (2.65)***	.23 (4.66)***	.25 (8.04)***
Cultural diffusion	.31 (2.41)***	.20 (1.63)^†^	
Global exchange	.11 (.96)^†^		
Secular values		.34 (4.26)***	.57 (9.81)***
East Asia (dummy)			−.17 (−5.05)***
Adjusted R^2^	.72	.77	.82
N (societies)	81	81	84

Entries are unstandardized regression coefficients with their *T* values in parentheses. Test statistics of heteroskedasticity (White-test) and multicollinearity (variance inflation factors) reveal no violation of OLS assumptions. However, the DFFITs identify China as an influential case (outlier). Removing it, increases the explained variance by 2 to 3 percent in Models 1 and 2 and elevates the *T* value of Secular Values above that of Civic Entitlements

Significance levels: * *p* < .100;  ** *p* < .050; *** *p* < .005; ^†^ not significant (*p* > .100)

Global exchange, by contrast, shows no effect under control of the other variables and is replaced with secular values in model two. Doing so increases the explained variance from seventy-two to seventy-seven percent. It now turns out that secular values associate with the steepest gain in emancipative values while life opportunities turn insignificant. Thus, secular values mediate the effect of life opportunities: these opportunities contribute to emancipative values only insofar as they give rise to secular values. Still, our sample includes the distinct group of East Asian societies in which secular values are—in contrast to everywhere else—*not* indicative of an emancipatory culture. Ignoring the East Asian irregularity partly obscures the effect of secular values. By contrast, when we include an East Asia dummy^12^ to control for this irregularity, the effect of secular values surfaces more strongly. This is evident from model 3.^13^

**Fig. 3:**
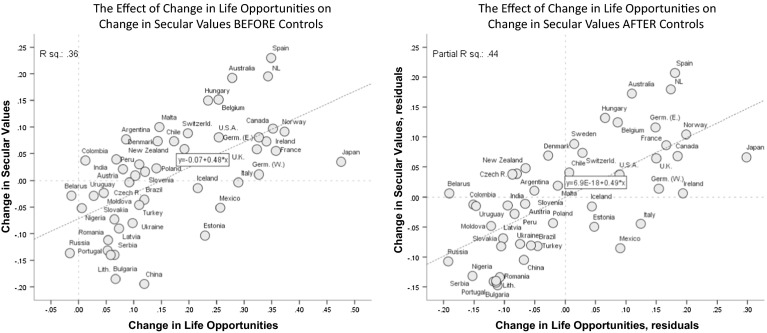
The dynamic relationship between change in life opportunities and change in secular values. Note: The analyses are limited to societies with an at least 10 year time distance between the earliest and latest survey of the WVS. Right-hand diagram controls the relationship for the start-level of secular values

Establishing purely cross-sectional associations is insufficient for a causal interpretation. Causality involves a dynamic relationship in which *change* in an outcome variable associates with *change* in its presumed treatment. This leads us to our second key finding: societies that made bigger progress towards permissive conditions also experienced larger gains in emancipative values (Fig. 3).

### Dynamic Evidence

We focus on the fifty societies for which we can measure change over ten or more years. On this basis, Appendix-Table 5 (OA 12: 23) correlates change in emancipative values with change in those permissive conditions that turned out as the strongest cross-sectional correlates in Table 1.

The longitudinal evidence is more selective than the cross-sectional one: fewer variables correlate with emancipative values in a dynamic way. Indeed, only secular values, life opportunities and cultural diffusion retain in the dynamic perspective the positive association with emancipative values. Civic entitlements, by contrast, do *not* correlate with emancipative values in a dynamic way. Hence, the association of civic entitlements with these values in the cross-section lacks a dynamic underpinning. It cannot be causal for this reason.

The analyses in Table 2 re-examine the bivariate results from Appendix-Table 3 in a multivariate framework, using dynamic regressions. We regress emancipative values at the time of the latest survey *T*_2_ on (1) themselves at the time of the earliest survey *T*_1_ and (2) on change from *T*_1_ to *T*_2_ in the treatment variables. This model is dynamic because, under control of the lagged dependent variable, we see for other predictors in the model how much they *shift* emancipative values at *T*_2_ upward or downward from where they were at *T*_1_. The other reason why the model is dynamic is that the predictors themselves measure change. Hence, the regression models show to what extent change from *T*_1_ to *T*_2_ in a given treatment shifts emancipative values at *T*_2_ upward or downward from their level at *T*_1_.

**Table 2:** Dynamic models explaining the shift in emancipative values from the earliest to latest survey with change in predictor variables

Predictors	Dependent variable: Emancipative values at *T*_2_	
	Model 1	Model 2
Constant	−.02 (.28)^†^	.05 (.94)^†^
Emancipative values at *T*_1_	.85 (4.71)***	.84 (5.25)***
Δ (*T*_2_ − *T*_1_) Life opportunities (sq.)	.28 (2.37)**	
Δ (*T*_2_ − *T*_1_) Civic entitlements	−.01 (−.14)^†^	.04 (.75)^†^
Δ (*T*_2_ − *T*_1_) Cultural diffusion	.27 (2.20)**	.18 (1.82)*
Δ (*T*_2_ − *T*_1_) Secular values		.58 (4.19)***
East Asia (dummy)		−.03 (−.63)^†^
Adjusted R^2^	.60	.69
*N* (societies)	47	49

Entries are unstandardized regression coefficients with their *T* values in parentheses. Test statistics of heteroskedasticity (White-test) and multicollinearity (variance inflation factors) reveal no violation of OLS assumptions. Influential case diagnostics (DFFITs) identify China as a leverage case. Excluding China, the coefficient for change in Secular Values drops somewhat (*b* = .58) and so does the *T* value (3.71) but it remains the most significant and strongest effect

Significance levels: * *p* < .100; ** *p* < .050; *** *p* < .005; ^†^ not significant (*p* > .100)

*T*_2_: Time of latest survey if at least 10 years after first survey (15 surveys from WVS round 4 with modal year 2000 and 37 surveys from round 5 with modal survey year 2006; mean year of *T*_2_ is 2004)

*T*_1_: Time of earliest survey if at least 10 years before last survey (23 surveys from WVS round 1 with modal survey year 1982, 22 surveys from round 2 with modal survey year 1990 and 7 surveys from round 3 with modal survey year 1996; mean year of *T*_1_ is 1987)

Δ (*T*_2_ − *T*_1_): Minimum time distance is 10 years, maximum is 27 years, mean time distance is 17 years

Including the lagged dependent variable among the predictors has some more desirable properties. For one, we reduce the problem of endogeneity: should other predictors in the model be endogenous to emancipative values, the lagged level of emancipative values absorbs this endogeneity. Next, we reduce omitted variable bias: lagged emancipative values embody virtually every prior influence on these values, including influences we are unaware of (Pascarella and Wolniak 2004).^14^

Under these premises, the two models in Table 2 show that emancipative values are self-perpetuating over time. This is evident from the strong influence of lagged emancipative values. Beyond that, an increase from *T*_1_ to *T*_2_ in life opportunities by one unit shifts emancipative values at *T*_2_ upward from its level at *T*_1_ by a .28-unit. A one-unit increase in the mean level of emancipative values in other societies of the same culture zone elevates these values by another .27-unit. Expanding civic entitlements show *no* effect on rising emancipative values, confirming the lack of a dynamic association in Appendix-Table 3.

If we replace change in life opportunities with change in secular values (model 2), the explained variance increases from sixty to sixty-nine percent. Expanding civic entitlements remain insignificant and cultural diffusion drops in significance. Changing secular values is now by far the strongest predictor: a one-unit rise in secular values yields a .58-unit rise in emancipative values. Interestingly, while the East Asia dummy was significant in the cross-section, it no longer is in the dynamic perspective. Thus, East Asian traditions are linked with a low *level* of emancipative values, but they do not hamper the *rise* of emancipative values from that low level.

These findings suggest that the rise of emancipative values is *not* induced by improvements in institutional conditions but in existential conditions. And this process is mediated by secular values: ascending life opportunities give rise to emancipative values mostly because this ascension strengthens secular values.

**Fig. 4:**
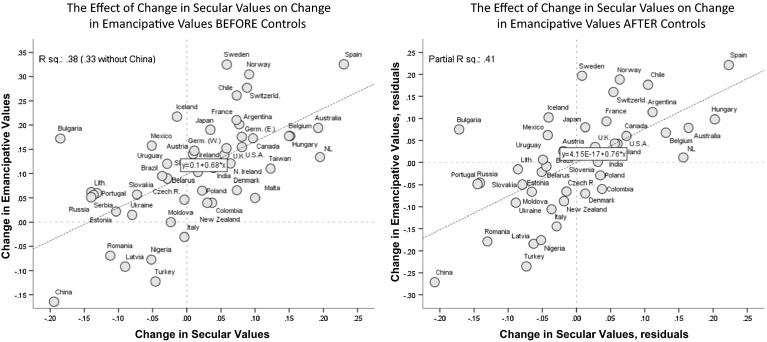
The dynamic relationship between emancipative values and secular values. Note: Analysis is limited to societies with an at least 10 years time distance between the earliest and latest survey in the WVS. *Right-hand* diagram is thepartial regression plot of model 2 in Table 5

Figures 3 and 4 visualize the sequential dynamic. Figure 3 illustrates the effect of ascending life opportunities on rising secular values; Fig. 4 illustrates the effect of rising secular values on rising emancipative values. In both figures, the left-hand diagram shows the uncontrolled effect, while the right-hand diagram shows the effect under controls.

The left-hand diagram of Fig. 3 shows that societies with the lowest ascension of life opportunities—namely post-Soviet societies such as Russia, Belarus and Ukraine—experienced a decrease in secular values. Conversely, societies with some of the highest ascensions of life opportunities—including Spain, Ireland and Norway—also experienced the largest increases in secular values.

China is a pronounced outlier from this logic, together with Japan: in both cases, change towards secular values is considerably lower than the ascension of life opportunities suggests. Partly, these two societies’ deviation from the close relationship between ascending life opportunities and rising secular values is explained by the fact that they both started from an unusually high level of secular values: they couldn’t get much more secular, even with massively improving living opportunities. Thus, when we control for the start-level of secularism (see right-hand diagram of Fig. 3), China and Japan move closer to the regression line and the explained variance improves from thirty-six to forty-four percent.

In Fig. 4, China appears as a leverage case at the opposite end of Spain. Thus, these two societies provide a particularly illustrative contrast as concerns the dependence of rising emancipative values on parallel gains in secular values. Again, the post-Soviet societies are illustrative too: with the resurgence of religiosity after a failed secular doctrine, secular values declined, which apparently prevented the rise of emancipative values.

### Cohort-Related Evidence

Our third key finding is that cohorts whose members grew up at times with more permissive conditions exhibit stronger emancipative values today than members of cohorts whose upbringing was characterized by more restrictive conditions.

**Fig. 5:**
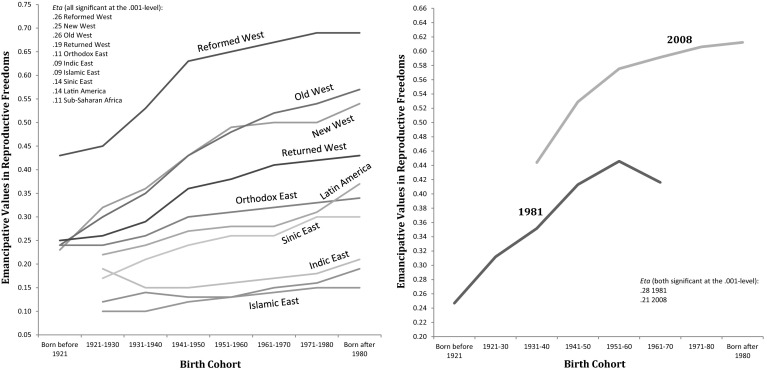
The cohort-pattern in emancipative values with regard to sexual freedoms. Note: For classification of societies into the culture zones of the* left-hand* diagram, see OA 1 (p. 6).* Right-hand* diagram limited to societies that participated in both the earliest and latest round of the WVS: Australia, Canada, France, West Germany, Italy, Japan, The Netherlands, Norway, Sweden, USA, UK. National samples are weighted to equal size (N = 1,000).

To demonstrate this point, the left-hand diagram of Fig. 5 plots the level of emancipative values for the members of eight successive cohorts, separately for Welzel’s (2013: 23) ten global culture zones (see OA 2, Appendix-Table 2, p. 5). There is an obvious tendency that emancipative values increase along the cohort succession in each culture zone. But the level of the tendency differs between culture zones, and so does its gradient. The level is lowest and the gradient flattest in the Islamic East, Sub-Saharan Africa and the Indic East—the zones with the most constrained life opportunities. Conversely, the level of the cohort trend is highest and the gradient steepest in the Western world—the part of the globe with the most abundant life opportunities. Ex-communist societies, Latin America and the Sinic East sit in between—as they are in terms of life opportunities.

The cohort pattern could reflect a lifecycle effect: younger people are always more emancipatory but become less so as they age. The right-hand diagram of Fig. 5, however, disconfirms a lifecycle effect. It shows how the level of emancipative values changed in each cohort among the dozen societies for which we have longitudinal evidence from the first to the fifth wave of the WVS, covering almost 30 years. Obviously, birth cohorts did not become less emancipatory as they aged. On the contrary, they became more emancipatory. Yet, they did become more emancipatory in ways that *reproduce* the cohort differences from almost 30 years before. Thus, even though the time trend strengthens emancipative values, the fact that it elevates each cohort from its specific start-level reproduces cohort differences over time.

**Fig. 6:**
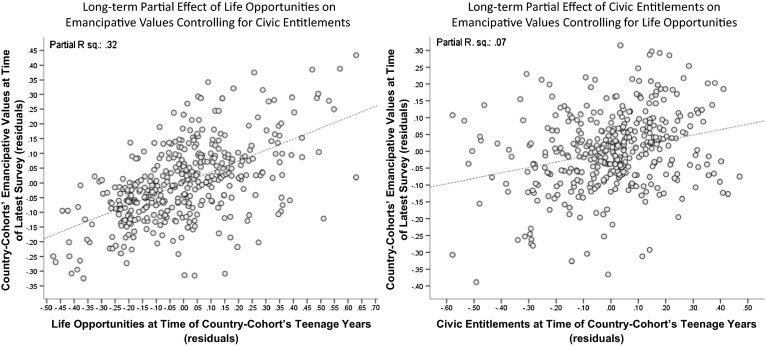
Predicting the emancipative values of country-cohorts by conditions during these cohorts’ teenage years. Note: Unit of analysis are country-cohorts (6 cohorts per 85 societies equals 510 observation units). Life opportunities proxied by Vanhanen data on the combined literacy and urbanization rate; civic entitlements proxied by Vanhanen’s democratization index (see OA 13–15, pp. 24–27)

Figure 6 shows two scatterplots obtained from regressing the country-cohorts’ emancipative values today on life opportunities and civic entitlements prevalent in the given country at the time of the respective cohort’s upbringing.^15^ The evidence is clear: cohort members exhibit stronger emancipative values today, the more permissive the conditions of their country were at the time of their upbringing. However, among the two permissive conditions, life opportunities are more important than civic entitlements: the former explains thirty-two percent, the latter only seven percent of the country-cohorts’ emancipative values (in total, we explain sixty-eight percent, so the remainder of twenty-nine percent is due to inseparable overlap between life opportunities and civic entitlements).^16^

The impact of life opportunities remains significant when we clean it from endogeneity to emancipative values. To do so we isolate the part of life opportunities that is unexplained by the emancipative values of the cohorts grown up *before* these opportunities. These “endogeneity-clean” life opportunities retain a significant and positive effect on the emancipative values of the cohorts grown-up *under* these opportunities. By contrast, “endogeneity-clean” civic entitlements entirely loose significance: de-coupled from the emancipative values of the cohorts grown-up *before* these entitlements, they show no more influence on the emancipative values of the cohorts grown-up *under* these entitlements. These findings suggest that the emancipatory effect of formative life opportunities is real, while that of formative civic entitlements is not.

There is again evidence for the role of secular values as a mediator between life opportunities and emancipative values: (1) life opportunities during the decade of a cohort’s upbringing explain thirty-eight percent of this cohort’s secular values today^17^; (2) secular values during the decade of a cohort’s upbringing explain thirty percent of this cohort’s emancipative values today.^18^ In other words, while preceding life opportunities explain subsequent secular values, preceding secular values explain subsequent emancipative values. We find no effects in the opposite direction of impact. These regression results are documented in OA 17 (p. 31).

### Multilevel Evidence

This section evidences the micro-foundation of our findings. As we will see, individuals who experience richer opportunities tend to be more emancipatory in their orientation. However, opportunity endowments strengthen people’s emancipative values more by the social *sharedness* of these endowments than by their individual uniqueness.

The multilevel regressions in Table 3 demonstrate these points. The societal-level component of the three models confirms the results of the cross-sectional regressions in Table 1. The novel part are the individual-level effects, all of which point in the expected direction: emancipative values in fields other than sexuality, together with secular values, show the strongest effects on support for sexual freedoms. However, characteristics of the society in which a person lives strengthen this person’s emancipative values more than her own characteristics.^19^ For instance, a one-unit increase in a person’s own secular values strengthens this person’s emancipative values by a .18-unit, but the *same* increase in the social prevalence of secular values strengthens this person’s emancipative values by a .46-unit. In short, the part of one’s secularism that one has in common with most others in one’s society strengthens one’s emancipative values more than the part that exceeds the secularism of most others.

**Table 3:** Multi-level models explaining individual respondents’ emancipative values

Predictors (at time of latest survey)	Dependent variable: emancipative values with regard to sexual freedoms (latest survey, 2000–2008)		
	Model 1	Model 2	Model 3
Constant	.32 (32.19)***	.33 (39.52)***	.32 (38.75)***
Societal-level effects (SL)			
Civic entitlements	.17 (3.20)***	.19 (5.21)***	.22 (6.89)***
Life opportunities (sq.)	.34 (5.66)***	.09 (1.71)*	
Secular values		.46 (5.50)***	.52 (7.45)***
East Asia (dummy)	−.09 (−2.95)**	−.15 (−4.31)***	−.14 (−4.02)***
Individual-level effects (IL)			
Female sex	.02 (8.42)***	.02 (9.76)***	.02 (9.74)***
Birth year	.13 (9.79)***	.13 (9.86)***	.13 (9.83)***
Formal education	.09 (13.68)***	.09 (13.83)***	.09 (13.89)***
Other emancipative values	.18 (11.66)***	.18 (11.67)***	.18 (11.67)***
Secular values	.18 (15.23)***	.18 (15.24)***	.18 (15.24)***
Cross-level interactions			
Female (IL) × Entitlements (SL)	.01 (.89)^†^	.02 (1.53)^†^	.02 (1.98)*
Female (IL) × opportunities (SL)	.06 (4.60)***	.00 (.24)^†^	
Female (IL) × secular values (SL)		.11 (5.82)***	.11 (7.38)***
Female (IL) × East Asia (SL)	−.01 (−.95)^†^	−.02 (−2.61)**	−.02 (−2.56)**
Birth year (IL) × entitlements (SL)	.18 (2.92)**	.19 (2.99)***	.18 (3.78)***
Birth year (IL) × Opportunities (SL)	.02 (.22)^†^	−.03 (−.23)^†^	
Birth year (IL) × secular Values (SL)		.08 (.72)^†^	.06 (.80)^†^
Birth Year (IL) × East Asia (SL)	−.00 (−.10)^†^	−.02 (−.33)^†^	−.02 (−.36)^†^
Education (IL) × entitlements (SL)	.11 (3.40)***	.12 (3.78)***	.12 (4.93)***
Education (IL) × opportunities (SL)	.08 (1.90)*	−.01 (.22)^†^	
Education (IL) × secular values (SL)		.12 (2.08)**	.13 (3.02)***
Education (IL) × East Asia (SL)	−.00 (.04)^†^	−.02 (−.78)^†^	−.02 (−.77)^†^
Error reduction (%)			
IL-variation of DV	13.0 %	13.0 %	13.0 %
SL-variation of DV	73.1 %	82.0 %	81.6 %
Female’s IL-effect	42.5 %	60.0 %	60.0 %
Birth year’s IL-effect	24.3 %	23.7 %	25.1 %
Education’s IL-effect	48.2 %	48.5 %	49.2 %
*N* (observations)	132,099 individuals in 89 societies		

Entries are unstandardized regression coefficients with T-ratios in parentheses (based on robust standard errors). Models calculated with HLM 6.02. Samples weighted to equal size, using the latest survey from each society (2000–2008). Reduction of error calculated from change in random variance component relative to the empty model. All individual-level variables (except female sex) are country-mean centered; societal-level variables (except East Asia dummy) are global-mean centered. 64 % of the variation in emancipative values is at the individual level, 36 % at the societal level (i.e., intra-class correlation: .60)

Significance levels: * *p* < .050; ** *p* < .010; *** *p* < .001; ^†^ not significant (*p* > .050)

**Fig. 7:**
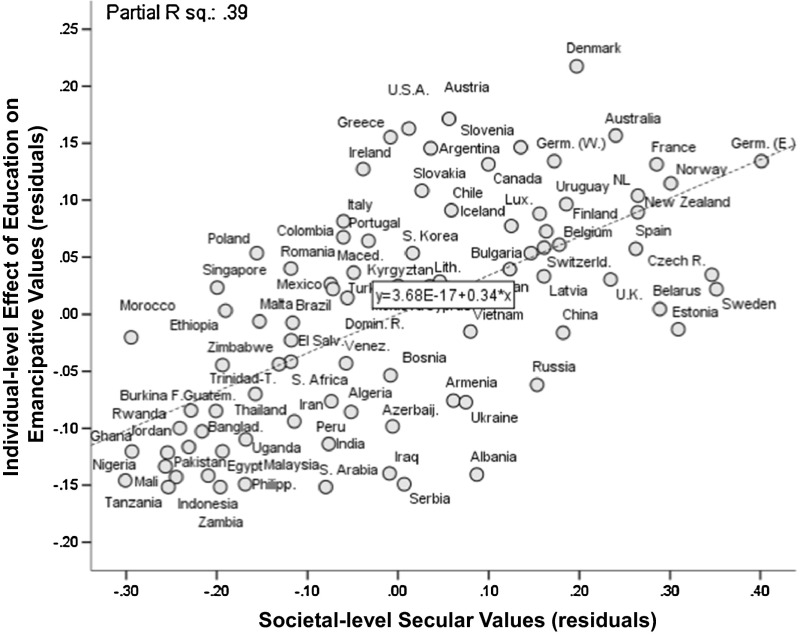
Variation in education’s individual-level effect on emancipative values as a function of secular values at the societal level

Moreover, shared secular values enhance the emancipatory effect of education. This is evident from Fig. 7 and the cross-level interactions in Table 3: an individual’s education strengthens her emancipative values more pronouncedly when this education takes place in a more secular society.^20^

## Discussion

Emancipative values in the domain of sexuality vary greatly across societies around the globe. Contrary to the notion that culture is a constant, these values have been changing considerably, showing sizeable increases in the overwhelming majority of societies, albeit within differing ranges of growth. Levels as well as changes in emancipative values co-vary strongly—and meaningfully—with objective social indicators, especially those measuring life opportunity endowments on a mass scale. In general, emancipative values are more prevalent where life opportunities are more abundant for large population segments. Correspondingly, emancipative values have grown most rapidly where life opportunities have been ascending on the steepest slope.

However, in the domain of sexuality the emancipatory effect of ascending life opportunities is indirect: abundant life opportunities favor emancipative values in sexual matters only in as far as their ascension diminishes the appeal of religion and, hence, gives rise to secular values. In the process of sexual emancipation, secularization is a necessary intermediary step because religion is the most powerful preservative of traditional sex norms.

Our global, cross-cultural evidence shows that people’s subjective values are in touch with objective utilities. This reality link allows moral systems to evolve “naturally” in response to shifts in objective utilities. If these shifts move upward the freedom ladder, emancipative values rise. From an evolutionary point of view, emancipative values are a true moral innovation because their rise breaks a perennial limitation of human morality: the age-old consensus over traditional sex norms. In a humanitarian sense, sexual emancipation might even be characterized as ethical “progress,” for emancipative values associate with more trust in strangers, stronger civic activism, wider circles of solidarity and less tolerance of discrimination and human casualties (Deutsch and Welzel 2011; Inglehart et al. 2015; Welzel and Delhey 2015).

Unfortunately, we have no direct observation of the process generating value change between two measurements in time. Here we face a black box that we cannot unpack in the absence of panel data. And in the absence of experimental control, the causal status of our findings inevitably retains a speculative element. The best we can do to lend further credibility to our insights is to continue the time series and produce a richer longitudinal database for additional examinations. The WVS remains the most important tool for this purpose. Its continuation and extension into uncovered areas needs to rank high on the research agenda of value change.

## Electronic supplementary material

Below is the link to the electronic supplementary material.

Supplementary material 1 (DOCX 133 kb)

## Footnotes

1 To the recentness of this development, see the Response Section in our Online Appendix (OA 23: 42) at this journal's website (http://www.springer.com/social+sciences/journal/11205).

2 An exception is the US for which the richest longitudinal evidence in population attitudes exists. In line with our claims, Twenge et al. (2015b) demonstrate a pronounced generational increase in sexual tolerance among US residents. This trend is paralleled by a similarly pronounced decline in religiosity (Twenge et al. 2015a). Our article shows that these intertwined trends apply to much wider parts of the world than just the US.

3 The utility ladder of freedoms coincides with the hierarchy of motivations: higher rungs on the utility ladder correspond with higher-ordered motivations (Maslow 1954). Specifically, the more evolved drive for self-development and learning lends itself more closely to freedoms than the more basic drive for defense and survival (Lawrence and Nohria 2002). Yet, the emphasis of the utility ladder on *shared* utilities gives the utility ladder a *social* dimension that the hierarchy of motivations lacks. For this reason, the utility ladder is a more adequate tool to describe *social* change than the hierarchy of motivations.

4 For a discussion of inter- and intra-societal differences in human values, see the Response Section in our Online Appendix (OA 23: 39, 41) at this journal's website (http://www.springer.com/social+sciences/journal/11205).

5 Using the pooled individual-level data, including the latest available survey from each of our ninety-three societies (*N* = 160,000 respondents), the Cronbach’s alpha for the three items of emancipative values in the field of reproductive freedoms is .80, which compares to .64 for the three items of gender equity, .35 for the three items of personal autonomy and .40 for the three items of people’s voice. What is more, emancipative values in the field of reproductive freedoms correlate at *r* = .31 with participation in nonviolent social movement activity, .23 with the perception of one’s daily tasks as intellectual and .38 with an enlightened understanding of democracy. The associations with these expected correlates of emancipative values are .22, .19 and .37 for an emphasis on gender equity; .21, .18 and .19 for an emphasis on personal autonomy; and .24, .15 and .22 for an emphasis on people’s voice.

6 Since we are concerned with change in variables that move at glacial pace, it is important to measure change over the largest available time interval. Short-term changes, by contrast, easily capture random fluctuations that are irrelevant in a developmental context. Throughout this article, change in a given variable is calculated by subtracting the earliest score from its latest score. Since all variables are standardized into a range from 0 to 1, changes range from a theoretical minimum of -1 to a theoretical maximum of +1, with 0 indicating no change. Against skepticism in the early literature (e.g., Cronbach and Furby 1970), recent scholarship defends the analyses of change scores as essential to the understanding of dynamic relations (Liker et al. 1985; Allison 1990; Miller and Kane 2001). For an exemplary analysis of change scores in the field of women’s empowerment, see Alexander (2012).

7 These cultural changes are paralleled by a significant decrease in people’s willingness to fight for their country in the case of war (Inglehart et al. 2015).

8 For a further discussion of this index, see the Response Section in OA 23 (p. 37).

9 We keep indicators of permissive conditions separate across the existential, institutional and cultural domains in order to uncover these domains’ distinct impacts.

10 Alternatively, we used the “good governance” indicators from the World Bank (Kaufman et al. 2010). But these indicators show no closer association with emancipative values than do the indicators of the Risk Guide.

11 We experimented with different prevalence measures, using the median instead of the mean or the geometric and quadratic means instead of the arithmetic means as well as proportions of respondents scoring in the upper quartiles and quintiles of our multi-point value measures. None of these variants produced stronger results than those obtained by the arithmetic population means. For a discussion of the appropriateness of aggregating individual-level values to the society level, see the Response Section in OA 23 (pp. 39–41).

12 China, Hong Kong, Japan, South Korea, Taiwan, Thailand, and Vietnam are coded 1. All other societies are coded 0.

13 For a conceptual discussion and empirical treatment of endogeneity, see the Response Section in OA 23 (p. 40).

14 Against criticism by Achen (2001), Keele and Kelly (2006) defend the use of lagged dependent variables as appropriate for most cases of application.

15 OA 17 (p. 28) documents the regression results.

16 The explanatory power of life opportunities over emancipative values is as strong for the cohort differences within the same countries (i.e., 64 % explained variance) as it is for the country differences within the same cohorts (58 %). Also, the spatial relatedness of different cohort observations from the same countries and the temporal relatedness of different country observations from the same cohorts apply to both emancipative values and its predictor variables. Spatial and temporal correlation is, thus, the same on both sides of the equation, which assures comparability.

17 The effect exists controlling for civic entitlements during the decade of a cohort’s upbringing, which explain only an insignificant one percent of this cohort’s secular values today.

18 The effect exists controlling for life opportunities during the cohort’s upbringing, which explain twelve percent of this cohort’s emancipative values today.

19 This conclusion is safe because all individual-level variables are centered on their societal mean level. This filters out from the individual-level measures all overlapping variance with similar societal-level measures. Thus, we perfectly separate individual-level and societal-level effects.

20 There are more cross-level interactions to report but these are beyond the focus of this article, so we refrain from stating them here. Let us just briefly mention that the emancipatory effect of being female greatly enhances when secular values are more widespread in a society.
