# Supplementary material for: Emancipating Sexuality: Breakthroughs into a Bulwark of Tradition
Source: Soc Indic Res. 2015 Oct 28;129(2):909–35. doi: 10.1007/s11205-015-1137-9 (PMC5080327; doi:10.1007/s11205-015-1137-9)
Supplement: Supplementary file 1 — Supplementary material 1 (DOCX 133 kb) [file 11205_2015_1137_MOESM1_ESM.docx]

ONLINE APPENDIX (OA)

Alexander, A.C., R. Inglehart & C. Welzel (2015): “Sexual Emancipation: Breakthroughs into a Bulwark of Tradition.” *Social Indicators Research* 102: forthcoming.

# CONTENT

CONTENT 1

Preliminary Remark 3

OA 1: Emancipative Values in Reproductive Freedoms 4

Appendix-Table 1. Cross-Cultural Reliability and Equivalence of the Three Emancipative Values Items 6

OA 2: Emancipative Values in Other Domains 7

OA 3: Secular Values 10

Appendix-Table 2. Cross-Cultural Equivalence of the Components of Secular Values 12

OA 4: Descriptive Statistics for Different Components of Emancipative Values 13

OA 5: Civic Entitlements Index 14

OA 6: Life Opportunities Index 15

Appendix-Table 3. Factor Analysis Justifying the Life Opportunities Index 16

OA 7: Global Exchange Index 17

OA 8: Cultural Diffusion Index 18

OA 9: Institutional Functioning Index 19

OA 10: Additional Societal-level Variables 20

OA 11: Cross-Sectional Societal-level Correlations with Emancipative Values 22

Appendix-Table 4. Cross-sectional Correlates of Emancipative Values in Reproductive Freedoms 22

OA 12: Dynamic Societal-level Correlations with Emancipative Values 23

Appendix-Table 5. Longitudinal Correlates of Rising Emancipative Values in Reproductive Freedoms 23

OA 13: Proxy for Life Opportunities in the Cohort Analysis 24

OA 14: Proxy for Civic Entitlements in the Cohort Analysis 25

OA 15: Emancipative Values in the Cohort Analysis 26

Appendix-Table 6. Decennial Attribution Scheme 26

OA 16: Results from Multiple Imputations and ‘SUR’ 27

OA 17: Temporally Ordered Panel Regressions with Country-Cohorts 28

OA 18: Additional Individual-level Data for the Multi-level Models 29

OA 19: Temporal Distances per Society for the Change Measures 30

Appendix-Table 9. Variability of Key Variables in the Longitudinal Sample: Standard Deviation (Range) 30

OA 20: Descriptive Statistics for the Cross-Sectional and Dynamic Analyses 31

OA 21: Descriptive Statistics for the Country-Cohort Analyses 35

OA 22: Descriptive Statistics for the Multi-level Analyses 36

OA 23: Response Section 37

REVIEWER #1 37

REVIEWER #3 42

# Preliminary Remark

All data are available for replication analyses, summarized in four datasets, one for each of the analytical steps in the article. To obtain the data, please send an e-mail to the corresponding authors, Amy C. Alexander or Christian Welzel (amy.catherine. alexander@gmail.com; cwelzel@gmail.com). Descriptive statistics for all variables used in this study are available the the end of the appendix, at OA 20 to OA 22 (pp. 30-35).

# OA 1: Emancipative Values in Reproductive Freedoms

Emancipative values are operationalized as one of the four sub-components of Welzel’s (2013) encompassing index of emancipative values. The encompassing measure of emancipative values is a multi-point index from minimum 0 to maximum 1.0 based on twelve items from the World Values Surveys (World Values Survey Association 2010). The World Values Surveys have been conducted in five waves in more than 90 countries around the globe. Samples represent the adult residential population of a country (people at and above 18 years of age), with sample sizes averaging at 1,200 respondents per country. Interviews are based on a fully standardized master questionnaire, translated (with back-translation checks) and pre-tested in local languages. Details on fieldwork, questionnaire, sampling methods, and data are available online at: www.worldvaluessurvey.org.

The countries sampled by the World Values Surveys represent almost 90 percent of the world population and include the countries with the largest populations and biggest economies in each world region. They cover the full range of variation in cultural traditions, levels of development and political regimes that exists in the world.

As defined by Welzel (2013), emancipative values idolize a life free from external domination, for which reason these values emphasize equal freedoms for everyone. Thus, emancipative values involve a double emphasis on freedom of choice and equality of opportunities. Screening the World Values Surveys for items that have been fielded repeatedly, Welzel identified twelve items that represent an emphasis on freedom of choice or equality of opportunities or both. Three of these merge into the ‘*choice*’ index, which is one of four constitutive components of emancipative values. The choice index measures emancipative values in the domain of reproductive freedoms, indicating people’s support for abortion, divorce and homosexuality as tolerable. Below follows the question wording and a syntax for the coding procedures used to create the index of emancipative values in the field of reproductive freedoms.

*Question Wording* [variable numbers as in the master questionnaire of WVS wave 5]:

Please tell me for each of the following actions whether you think it can always be justified, never be justified, or something in between using this card:

| V202. Homosexuality | 1 | 2 | 3 | 4 | 5 | 6 | 7 | 8 | 9 | 10 |
| --- | --- | --- | --- | --- | --- | --- | --- | --- | --- | --- |
| V204. Abortion | 1 | 2 | 3 | 4 | 5 | 6 | 7 | 8 | 9 | 10 |
| V205 Divorce | 1 | 2 | 3 | 4 | 5 | 6 | 7 | 8 | 9 | 10 |

Never Always

justifiable justifiable

Codes are rescaled from minimum 0 to maximum 1 for each of the three items. This is done by subtracting 1 from the respondent’s score and then dividing the resulting difference by 9 (given score minus minimum possible score divided by the difference between the maximum and minimum possible score). To create the choice sub-index, recoded scores are averaged over the three items. The syntax is as follows:

compute homolib=(v202-1)/(10-1).

recode homolib (sysmiss=-99).

mis val homolib (-99).

var lab homolib "homosex acceptable".

exec.

compute abortlib=(v204-1)/(10-1).

recode abortlib (sysmiss=-99).

mis val abortlib (-99).

var lab abortlib "abortion acceptable".

exec.

compute divorlib=(v205-1)/(10-1).

recode divorlib (sysmiss=-99).

mis val divorlib (-99).

var lab divorlib "divorce acceptable".

exec.

Appendix-Table 1 on the next page demonstrates that the uni-dimensionality and high scale reliability of the three items of emancipative values is a cross-cultural universal. Thus, there is sufficient equivalence in item functioning to consider the index of emancipative values a reliable and valid cross-cultural construct.

To obtain a societal-level measure of the prevalence of emancipative values in a society we calculate each national sample’s mean score on the emancipative values index and do this separately for each year in which the WVS has been conducted.

## Appendix-Table 1. Cross-Cultural Reliability and Equivalence of the Three Emancipative Values Items

| Culture Zones: | Cronbach’s Alpha^a)^ | Factor Loadings^b)^: | | | *N* (countries) |
| --- | --- | --- | --- | --- | --- |
|  |  | Homosexuality | Abortion | Divorce |  |
| Reformed West | .79 | .80 | .85 | .88 | 11,318 (8) |
| New West | .80 | .81 | .86 | .86 | 9,368 (6) |
| Old West | .83 | .83 | .88 | .88 | 17,075 (12) |
| Returned West | .77 | .75 | .87 | .87 | 15,996 (11) |
| Orthodox East | .71 | .64 | .87 | .86 | 24,012 (14) |
| Indic East | .77 | .80 | .87 | .83 | 14,205 (8) |
| Islamic East | .56 | .68 | .84 | .72 | 11,572 (6) |
| Sinic East | .80 | .80 | .85 | .88 | 9,532 (6) |
| Latin America | .66 | .79 | .76 | .77 | 19,786 (12) |
| Subs. Africa | .73 | .81 | .88 | .76 | 15,663 (11) |
| Mean | .74 | .77 | .85 | .83 |  |
| Variance Coeff. | .11 | .08 | .05 | .07 |  |
| *Notes*: Analyses conducted on the basis of the last survey from each country. Countries attributed to culture zones as follows: *Old West*: Andorra, Austria, Belgium, (Cyprus), France, (Greece), Ireland, (Israel), Italy, Luxembourg, Malta, Portugal, Spain; *Reformed West*: Denmark, Finland, Germany (West), Iceland, Netherlands, Norway, Sweden, Switzerland, U.K.; *New West*: Australia, Canada, New Zealand, U.S.A.; *Returned West*: Croatia, Czechia, Estonia, Germany (East), Hungary, Latvia, Lithuania, Poland, Slovakia, Slovenia; *Orthodox East*: Albania, Armenia, Azerbaijan, Belarus, Bosnia, Bulgaria, Georgia, Kyrgyzstan, Macedonia, Moldova, Romania, Russia, Serbia, Ukraine; *Islamic East*: Algeria, Egypt, Iran, Iraq, Jordan, Morocco, Saudi Arabia, Turkey; *Indic Asia*: Bangladesh, India, Indonesia, Malaysia, Pakistan, Philippines, Singapore, Thailand; *Sinic East*: China, Hong Kong, Japan, South Korea, Taiwan, Vietnam; *Latin America*: Argentina, Brazil, Chile, Colombia, Dominican Republic, El Salvador, Guatemala, Mexico, Peru, Venezuela, Trinidad and Tobago, Uruguay; *Sub-Saharan Africa*: Burkina Faso, Ghana, Mali, Nigeria, Rwanda, South Africa, Tanzania, Uganda, Zambia, Zimbabwe.  a) Scale reliability of a combination of the homosexuality, abortion and divorce items.  b) Loadings on first and single factor in a factor analysis over the country-pooled individual-level data per culture zone. | | | | | |
|  | | | | | |

# OA 2: Emancipative Values in Other Domains

The measure ‘other emancipative values’ uses the remaining three sub-components of Welzel’s encompassing measure of emancipative values, including (1) the ‘autonomy’ sub-index, (2) the ‘equality’ sub-index and (3) the ‘voice’ sub-index, each of which is based on three items. The ‘autonomy sub-index measures the respondents’ agreement with independence and imagination but not with obedience as desired child qualities. The ‘equality’ sub-index measures the respondents’ rejection of male priority in education, jobs and politics. The ‘voice’ sub-index measures the respondents’ priorities for freedom of speech and for people having a voice and a say in how things are done in their society. To measure these priorities, three items known from the concept of postmaterialism are used.

*Question Wording* [variable numbers in the wave-5 version of the WVS questionnaire]:

(1) *Autonomy Sub-Index*: Four-point index from 0 to 1.

“Here is a list of qualities that children can be encouraged to learn at home. Which, if any, do you consider to be especially important?

|  | Mentioned | Not mentioned |
| --- | --- | --- |
| V12. Independence | 1 | 2 |
| V15. Imagination | 1 | 2 |
| V21. Obedience | 1 | 2” |

Mentioning of ‘independence’ and ‘imagination’ are both coded 1 and 0 otherwise. Mentioning of ‘obedience’ is coded 0 and 1 otherwise. To create the autonomy sub-index, recoded scores are averaged over the three items.

(2) *Voice Sub-Index*: Six-point index from 0 to 1.

“People sometimes talk about what the aims of this country should be for the next ten years. On this card are listed some of the goals which different people would give top priority. Would you please say which one of these you, yourself, consider the most important? (…) And second most important?”

There are twelve aims in total, organized in three four-item batteries, with two postmaterialist items in each battery. Of these six postmaterialist items, three measure an emphasis on people’s voice. The first two of these three items listed below appear jointly in the second item battery (variables V71 for first priority and V72 for second priority); the third one appears separately in the first battery (variables V69 for first priority and V70 for second priority):

| - “Giving people more say in important government decisions” [code 2 in variables V71 and V72]  - “Protecting freedom of speech” [code 4 in variables V71 and V72]  - “Seeing that people have more say about how things are done at their jobs and in their communities”  [code 3 in variables V69 and V70]. |
| --- |

Responses are recoded into 0 when the item has not been chosen as important, 0.5 when it has been chosen as second most important and 1 when it has been chosen as most important. To create the index of voice values, the recoded scores are averaged over the three items.

(3) *Equality Sub-Index*: Twelve-point index from 0 to 1.

“Do you agree, disagree or neither agree nor disagree with the following statements? When jobs are scarce, men should have more right to a job than women.”

“For each of the following statements I read out, can you tell me how strongly you agree or disagree with each. Do you strongly agree, agree, disagree, or strongly disagree?

- A university education is more important for a boy than for a girl.

| - On the whole, men make better political leaders than women do.” |
| --- |

For the first item (V44), agree is coded 0, neither nor is coded .5 and disagree is coded 1. For the next two items (V62, V63), ‘strongly agree’ is coded 0, ‘agree’ is coded .25, ‘disagree’ is coded .75 and ‘strongly disagree’ is coded 1. To create the equality sub-index, recoded scores are averaged over the three items.

To create a summary measure of ‘other emancipative values’ over these three sub-indices, the respondents’ scores are simply averaged across the three sub-indices.

***SYNTAX***

***Sub-Index 1 (3 items): AUTONOMY***

recode v12 (1=1) (2=0) into indep.

recode indep (sysmiss=-99).

mis val indep (-99).

var lab indep "independ as kid qual".

exec.

recode v15 (1=1) (2=0) into imagin.

recode imagin (sysmiss=-99).

mis val imagin (-99).

var lab imagin "imagin as kid qual".

exec.

recode v21 (1=0) (2=1) into nonobed.

recode nonobed (sysmiss=-99).

mis val nonobed (-99).

var lab nonobed "obedience not kid qual".

exec.

compute autonomy=(indep+imagin+nonobed)/3.

exec.

***Sub-Index 2 (3 items): EQUALITY***

recode v44 (1=0) (2=.5) (3=1) into womjob.

recode womjob (sysmiss=-99).

mis val womjob (-99).

var lab womjob "gend equal: job".

exec.

recode v61 (1=0) (2=.33) (3=.66) (4=1) into wompol.

recode wompol (sysmiss=-99).

mis val wompol (-99).

var lab wompol "gend equal: politics".

exec.

recode v62 (1=0) (2=.33) (3=.66) (4=1) into womedu.

recode womedu (sysmiss=-99).

mis val womedu (-99).

var lab womedu "gend equal: education".

exec.

compute equality=(wompol+womedu+womjob)/3.

exec.

***Sub-Index 2 (3 items): VOICE***

if ((v71=2 and v72=4) or (v71=4 and v72=2)) voice1=1.

exec.

if ((v71=2 and v72 ne 4) or (v71=4 and v72 ne 2)) voice1=.66.

exec.

if ((v71 ne 2 and v72=4) or (v71 ne 4 and v72=2)) voice1=.33.

exec.

if ((v71 ne 2) and (v71 ne 4) and (v72 ne 2) and (v72 ne 4)) voice1=0.

exec.

recode voice1 (sysmiss=-99).

mis val voice1 (-99).

var lab voice1 "voice 1".

exec.

if (v69=3) voice2=1.

exec.

if (v70=3) voice2=.5.

exec.

if ((v69 ne 3) and (v70 ne 3)) voice2=0.

exec.

recode voice2 (sysmiss=-99).

mis val voice2 (-99).

var lab voice2 "voice 2".

exec.

compute voice=(voice1+voice2)/2.

recode voice (sysmiss=-99).

mis val voice (-99).

exec.

***Index ‘Other Emancipative Values: OEV***

compute oev=(autonomy+equality+voice)/3.

exec.

# OA 3: Secular Values

Secular values are measured using the ‘agnosticism’ sub-index of Welzel’s (2013) encompassing measure of secular values. This encompassing index measures secular values in the broader sense of distance to sacred sources of authority. In the context of the current study, there is a more specific interest in distance from the authority of religion in particular. This topic is addressed specifically by the agnosticism component of the encompassing measure. Thus, we measure secular values based on this particular sub-component. This sub-component is based on three items, one on the importance of religion, another one on whether someone is a religious person and yet another one on religious service.

The question on the importance of religion reads like this [variable V9 in the wave-5 version of the WVS questionnaire]:

“For each of the following, indicate how important it is in your life. Would you say it is (*read out and code one answer for each*):

|  |  | Very important | Rather important | Not very important | Not at all important |
| --- | --- | --- | --- | --- | --- |
| V9. | Religion | 1 | 2 | 3 | 4” |

Responses are recoded into 0 for ‘very important,’ .33 for ‘rather important,’ .66 for ‘not very important’ and 1 for ‘not at all important.’

The question on religious practice reads like this [V186 in the wave-5 version of the WVS questionnaire]:

“Apart from weddings and funerals, about how often do you attend religious services these days? (*Code one answer*):

1 More than once a week

2 Once a week

3 Once a month

4 Only on special holy days

5 Once a year

6 Less often

7 Never, practically never

(*NOTE: In Islamic societies, ask how frequently the respondent prays!*)”

Responses are recoded into a 7-point index from minimum 0 for ‘never, practically never’ to 1 for ‘more than once a week.’ This is done by subtracting 1 from all codes and dividing the resulting score by 6.

The question on religious self-perception [V187 in the wave-5 version of the WVS questionnaire] reads:

“Independently of whether you attend religious services or not, would you say you are (*read out and code one answer*):

1 A religious person

2 Not a religious person

3 An atheist

Responses are recoded into a dummy variable with code 0 for ‘a religious person’ and 1 for ‘not a religious person’ and ‘an atheist.’ To create the index of secular values, recoded responses to V9, V186 and V187 are averaged, yielding a multi-point index from 0 to 1.0.

***SYNTAX****

recode v9 (4=0) (3=.33) (2=.66) (1=1) into i_religimp.

recode i_religimp (sysmiss=-99).

mis val i_religimp (-99).

var lab i_religimp "inverse import of relig".

exec.

recode v187 (1=0) (2,3=1) into i_religbel.

recode i_religbel (sysmiss=-99).

mis val i_religbel (-99).

var lab i_religbel "inverse relig person".

exec.

recode v186 (1=0) (2=1) (3=2) (4=3) (5=4) (6=5) (7=6) into i_religprac.

compute i_religprac=i_religprac/6.

recode i_religprac (sysmiss=-99).

mis val i_religprac (-99).

var lab i_religprac "inverse relig practice".

exec.

compute SecVal = (i_religimp+i_relbel+i_relprac)/3.

exec.

To obtain a societal-level measure of the prevalence of secular values in a society we calculate each national sample’s mean score on the secular values index and do this separately for each year in which the WVS has been conducted.

## Appendix-Table 2. Cross-Cultural Equivalence of the Components of Secular Values

| Culture Zones: | Cronbach’s Alpha^a)^ | Factor Loadings^b)^: | | | *N* (countries) |
| --- | --- | --- | --- | --- | --- |
|  |  | Unimportance of Religion | Non-religious Person | Lack of Religious Practice |  |
| Reformed West | .73 | .86 | .80 | .81 | 11,318 (8) |
| New West | .80 | .89 | .82 | .86 | 9,368 (6) |
| Old West | .77 | .86 | .80 | .85 | 17,075 (12) |
| Returned West | .82 | .88 | .85 | .87 | 15,996 (11) |
| Orthodox East | .70 | .81 | .81 | .78 | 24,012 (14) |
| Indic East | .41 | .70 | .72 | .66 | 14,205 (8) |
| Islamic East | .33 | .66 | .73 | .62 | 11,572 (6) |
| Sinic East | .64 | .77 | .79 | .76 | 9,532 (6) |
| Latin America | .56 | .74 | .68 | .78 | 19,786 (12) |
| Subs. Africa | .58 | .66 | .77 | .77 | 15,663 (11) |
| Mean | .63 | .78 | .78 | .78 |  |
| Variance Coeff. | .27 | .12 | .06 | .11 |  |
| *Notes*: Analyses conducted on the basis of the last survey from each country. Countries attributed to culture zones as follows: *Old West*: Andorra, Austria, Belgium, (Cyprus), France, (Greece), Ireland, (Israel), Italy, Luxembourg, Malta, Portugal, Spain; *Reformed West*: Denmark, Finland, Germany (West), Iceland, Netherlands, Norway, Sweden, Switzerland, U.K.; *New West*: Australia, Canada, New Zealand, U.S.A.; *Returned West*: Croatia, Czechia, Estonia, Germany (East), Hungary, Latvia, Lithuania, Poland, Slovakia, Slovenia; *Orthodox East*: Albania, Armenia, Azerbaijan, Belarus, Bosnia, Bulgaria, Georgia, Kyrgyzstan, Macedonia, Moldova, Romania, Russia, Serbia, Ukraine; *Islamic East*: Algeria, Egypt, Iran, Iraq, Jordan, Morocco, Saudi Arabia, Turkey; *Indic Asia*: Bangladesh, India, Indonesia, Malaysia, Pakistan, Philippines, Singapore, Thailand; *Sinic East*: China, Hong Kong, Japan, South Korea, Taiwan, Vietnam; *Latin America*: Argentina, Brazil, Chile, Colombia, Dominican Republic, El Salvador, Guatemala, Mexico, Peru, Venezuela, Trinidad and Tobago, Uruguay; *Sub-Saharan Africa*: Burkina Faso, Ghana, Mali, Nigeria, Rwanda, South Africa, Tanzania, Uganda, Zambia, Zimbabwe.  a) Scale reliability of a combination of the three variables.  b) Loadings on first and single factor in a factor analysis over the country-pooled individual-level data per culture zone. | | | | | |
|  | | | | | |

# OA 4: Descriptive Statistics for Different Components of Emancipative Values

| **Statistics** | | | | | |
| --- | --- | --- | --- | --- | --- |
|  | | Emancipative Values: Reproductive Freedoms | Emancipative Values: Personal Autonomy | Emancipative Values: Gender Equity | Emancipative Values: People’s Voice |
| N | Valid | 163049 | 172378 | 142340 | 165985 |
|  | Missing | 10951 | 1622 | 31660 | 8015 |
| Mean | | .3140 | .4364 | .5584 | .3618 |
| Median | | .2593 | .3333 | .6067 | .3525 |
| Std. Deviation | | .28487 | .30881 | .27959 | .26852 |
| Minimum | | .00 | .00 | .00 | .00 |
| Maximum | | 1.00 | 1.00 | 1.00 | 1.00 |

# OA 5: Civic Entitlements Index

The following procedures describe Welzel’s (2013) measure of civic entitlements: the citizen rights index. The index is created in two steps. In the first step, we invert the two 1-to-7 scales for civil liberties and political rights by Freedom House (2012) (so that higher scores mean more freedom). Then we add the two scores and normalize the sum into a scale range from minimum 0 (no freedoms) to 1.0 (full amount of freedoms).^[[1]](#footnote-1)^ In the second step, the freedom scores are weighted down to the extent that they fail to tap human rights violations. The latter data are taken for the same years from the Cingranelli and Richards (CIRI) Human Rights Project (2010). We use the indices for non-repression of ‘physical integrity rights’ (an eight-point scale) and ‘empowerment rights’ (a ten-point scale), normalize them into a range from minimum 0 (maximum repression) to maximum 1 (minimal repression) and average the two scores.^[[2]](#footnote-2)^ These non-repression scores are then used as a weight for the freedom scores, using multiplication.^[[3]](#footnote-3)^ Thus, a society that has a high freedom score of, say, 0.80 but a lower non-repression score of, say, 0.60, obtains a final score of (0.60 * 0.80 =) 0.48 in civic entitlements. The emphasis of this measurement procedure is on civic entitlements that are truly respected in practice: what we have is not a latent variable of dimensionally related components but a *conditional* index that measures institutionalized freedoms to the extent to which they are respected in practice. Measures are taken from the same years as survey measures from the WVS. The index is validated in comparison with six other, most widely used measures of democracy by Welzel (2013: 256-277).

# OA 6: Life Opportunities Index

The idea behind this variable is to measure the richness of the opportunity endowments embodied in a society’s prevalent existential conditions. Thus, we look at socioeconomic and demographic indicators measuring how likely it is for the average person of a society to be materially well off, to live a long life filled with opportunities for intellectual development—opportunities that are not absorbed by raising many children. The following variables capture these opportunity endowments. Variables are measured repeatedly for each year for which we have a measure of emancipative values from the WVS.

*GDP* *per capita*: Per capita gross domestic product in purchasing power parities at constant US-Dollars in 2000. The highest income ($60,000 for Norway) is set at maximum 1.0 and all other figures are standardized towards this maximum ($54,000 would be .9 while $6,000 would be .1). Source: World Development Indicators Series (World Bank 2010).

*Income Equality*: Gini index for income inequality, standardized between minimum 0 and maximum 1.0 and then inverted (1 minus standardized index). Source: World Development Indicators Series (World Bank 2010).

*Life Expectancy at Birth*: Average life expectancy in years of a new born person, normalized into a scale range from minimum 0 to maximum 1.0. Source: World Development Indicators Series (World Bank 2010).

*Female Fertility Rate*: Mean number of child births per woman, normalized into a scale range from minimum 0 to maximum 1.0. Source: World Development Indicators Series (World Bank 2010).

*Tertiary Enrolment Ratio*: Percentage of the 20-25 year old in a country enrolled in tertiary-level education, normalized into a scale range from minimum 0 to maximum 1.0. Source: World Development Indicators Series (World Bank 2010).

*Years of Schooling*: The average person’s total years of primary, secondary, and tertiary-level school attendance in 2000, normalized into a scale range from minimum 0 to maximum 1.0. Source: Barro and Lee (2010).

Because opportunity endowments are strongly correlated across the various domains of human existence, these measures all reflect a single underlying dimension, that is, existential opportunities. This is demonstrated by the factor analysis in Appendix-Table 3, which justifies creating an encompassing summary measure of existential opportunities. This is done by adding the factor-weighted measures of the six components for each available year of measurement and then divide the summary score by the sum of the factor weights (to keep the resulting index within the score range from minimum 0 to maximum 1.0).

To straighten a skewed distribution on this index, we also operate with a squared measure of this index. The squared version of the index shows a more symmetric distribution: the skewness of the unsquared index is reduced from -.72 (a heavily right-skewed distribution) to +.21 (a slightly left-skewed distribution).

## Appendix-Table 3. Factor Analysis Justifying the Life Opportunities Index

| INDICATORS (at time of survey, 1981 - 2008): | FACTOR LOADING |
| --- | --- |
| Mean Number of Schooling Years | .89 |
| Life Expectancy at Birth | .87 |
| Female Fertility Rate (inverse) | .81 |
| Tertiary Enrollment Ratio | .74 |
| GDP per capita | .73 |
| Income Equality (inverse of Gini) | .56 |
| KMO | .71 |
| Explained Variance (fraction) | .60 |
| Cronbach’s Alpha | .85 |
| *N* (national surveys) | 241 |
| *Note*: Explorative factor analysis with one extracted factor (Kaiser-criterion) across the country and time pooled EVS/WVS, rounds 1 to 5 (1981 – 2008).  Summary factor scores on the underlying dimension extracted for each national survey, then standardized from minimum 0 to maximum 1.0 and squared. | |

# OA 7: Global Exchange Index

The global exchange index is based on Dreher, Gaston and Martens’ (2008) globalization index, which averages measures of economic, social, and political globalization in the following way (quote from their document):

**A. Economic Globalization [37%]**

i) • Actual Flows (50%)

• Trade (percent of GDP) (19%)

• Foreign Direct Investment, flows (percent of GDP) (20%)

• Foreign Direct Investment, stocks (percent of GDP) (24%)

• Portfolio Investment (percent of GDP) (17%)

• Income Payments to Foreign Nationals (percent of GDP) (20%)

ii) Restrictions (50%)

• Hidden Import Barriers (22%)

• Mean Tariff Rate (28%)

• Taxes on International Trade (percent of current revenue) (27%)

• Capital Account Restrictions (22%)

**B. Social Globalization [39%]**

i) • Data on Personal Contact (33%)

• Telephone Traffic (26%)

• Transfers (percent of GDP) (3%)

• International Tourism (26%)

• Foreign Population (percent of total population) (20%)

• International letters (per capita) (25%)

ii) Data on Information Flows (36%)

• Internet Users (per 1000 people) (36%)

• Television (per 1000 people) (36%)

• Trade in Newspapers (percent of GDP) (28%)

iii) Data on Cultural Proximity (31%)

• Number of McDonald's Restaurants (per capita) (43%)

• Number of Ikea (per capita) (44%)

• Trade in books (percent of GDP) (12%)

**C. Political Globalization [25%]**

• Embassies in Country (25%)

• Membership in International Organizations (28%)

• Participation in U.N. Security Council Missions (22%)

• International Treaties (25%)

We use these authors’ overall globalization measure (labelled global exchange index) as well as their separate measures for economic, social and political exchange and standardize each of these into a range from minimum 0 to maximum 1.0, with fractions for intermediate positions. Note that this standardization is based on the authors’ time-pooled database. Thus, the standardized index scores are temporally comparable: if a society has a higher score at a later point in time, this indeed indicates an absolute increase in its global linkages, not just a climb in relative position to other societies. Data are taken from the Quality of Governance Dataset at www.qog.se (Quality of Governance Institute 2010).

# OA 8: Cultural Diffusion Index

To create measures for the diffusion of emancipative values within culture zones, we attribute each society the mean score in emancipative values of all other societies of the same culture zone. The idea is that—because of culture-bound contagion--a given society’s score in emancipative values might partly be a function of the scores of other societies in the same culture zone. We calculated diffusion scores by treating each society as equally important, irrespective of its population size. Alternatively, we weighted each society’s score for the society’s population size, giving societies higher weights in proportion to their population size. This did not produce significantly different diffusion measures. Therefore, the article operates with the simpler diffusion measure.

For any variable, a culture zone diffusion measure is created by the following four steps:

(1) calculate the mean score in emancipative values for each culture zone,

(2) multiply each given culture zone’s mean score with the number of societies belonging to this zone,

(3) subtract from each product a given society’s own score in emancipative values,

(4) divide the difference for each culture zone by the number of societies in this zone minus 1.

Based on this procedure, each society receives a culture zone diffusion score that is *entirely exogenous* to this society’s own score in emancipative values. Since diffusion is about influxes *from outside* a given society, it is important to create diffusion scores such that they are indeed exogenous to the societies to which they are attributed.

# OA 9: Institutional Functioning Index

We transform the “Political Risk” indicator from the International Country Risk Guide (2010) into a score range from minimum 0 to maximum 1.0 such that lower scores indicate more and higher scores less political risk. This index provides a summary measure of various aspects of institutional functioning, indicating safety from the risk of public disorder, military takeover, corruption and confiscation. We take measures for each society separately for each year in which the WVS has been conducted. Data are taken from the Quality of Governance Database (Quality of Governance Institute 2010).

# OA 10: Additional Societal-level Variables

Measures for all of the following variables are taken from the same years in which the WVS has been conducted.

*Enduring Democracy*: ‘Democracy stock index’ measuring per society the historically accumulated experience with democracy until 1995, with a premium on more recent experience. Index is calculated by adding up the yearly -10 to +10 ‘autocracy-democracy’ scores of a country with a 1%-deflation rate for each year reaching back into the past. Thus, from the perspective of the year 1995, a hundred years back to 1895 scores cease to count. we normalized scores so that the minimum possible score is 0 and the maximum possible score is 1, with fractions of 1 for intermediate positions. Source: Gerring et al. (2005).

*External Peace*: Number of armed conflicts in which the government of a society has been involved since the end of World War II at time of the survey. Source: Gleditsch et al. (2002), UCDP/PRIO Armed Conflict Dataset-version 3 (online at www.pcr.uu.se /database/index.php).

*Internal Peace*: To measure the absence of internal violence and repression per society, we use Gibney, Wood and Cornett’s (2008) ‘political terror scale.’ Based on reports by Amnesty International and the US State Department, the scale measures the violation of citizen rights through state repression as well as terrorism by non-state actors. There are two highly inter-correlated scales for the two sources, each ranging from 1 (lowest repression level) to 5 (highest repression level). For each year in which the WVS has been conducted we calculate the average over the two scales, yielding a 9-point scale from 2 to 10 and inverted the measures into a range from minimum 0 (lowest internal peace) to maximum 1.0 (highest internal peace).

*Encompassing Peace*: Global peace index, inverted and normalized into a scale range from minimum 0 to maximum 1.0. Index measures the absence of inter-state and intra-state violence and conflict. Source: Vision of Humanity (online at: www.visionofhumanity.org/gpi-data/#/2010/scor).

*Tightness/Looseness*: Gelfand et al.’s (2011) tightness/looseness scores inverted so that scores increase from tightness to looseness and normalized into a scale range from minimum 0 (tightness pole) to maximum 1.0 (looseness pole).

*Collectivism/Individualism*: Index created in three steps from data published in the online supplement of Thornhill and Fincher et al. (2008) (see: http:// rspb. royalsocietypublishing.org/content/suppl/2009/03/20/275.1640.1279.DC1.html). First, we standardize individualism scores that Thornhill and Fincher et al. have taken from Hofstede (2001 [1980]) and Suh et al. (1998) into normalized scales from minimum 0 to maximum 1.0. Then we invert collectivism scores that Thornhill and Fincher et al. have taken from Gelfand et al. (2004) into individualism scores with the same standard scale range as the other two. Whenever all three measures are available, we take their average; otherwise we take the average of the remaining two or the score of the only available one. This is done to avoid losing a whole country when only one index is available. This procedure suggests that the three indices are inter-changeable, which is justified on the basis of very high inter-index correlations: *r* = .91 between Suh’s and Hofstede’s individualism scores (*N* = 45; *p* < .001, two-tailed); *r* = .85 between Suh’s individualism scores and Gelfand’s inverted collectivism scores (*N* = 38; *p* < .001, two-tailed); *r* = .75 between Hofstede’s individualism scores and Gelfand’s inverted collectivism scores (*N* = 46; *p* < .001, two-tailed).

*Consanguinity*: Consanguinity measures the average incidence of marriage within the wider family circle in a society; measures are logged to adjust a skewed distribution. Source: Woodley and Bell (2013).

*Patrilocality*: Percent married men per country (divided by 100) above 30 years of age living with their parents. Numbers calculated from latest available survey of the WVS.

# OA 11: Cross-Sectional Societal-level Correlations with Emancipative Values

## Appendix-Table 4. Cross-sectional Correlates of Emancipative Values in Reproductive Freedoms

| CORRELATES (latest survey: 2000 -2008): | EMANCIPATIVE VALUES (latest survey: 2000 -2008) |  |
| --- | --- | --- |
| Permissive *Existential* Conditions: |  |  |
| GDP per capita | .76*** (90) |  |
| Income Equality (inverse Gini) | .38*** (90) |  |
| Tertiary Enrollment | .61*** (89) |  |
| Schooling Years | .65*** (90) |  |
| Life Expectancy | .62*** (88) |  |
| Fertility Rate (inverse) | .48*** (89) |  |
| Existential Opportunities Index (sq.) | .79*** (87) |  |
| Permissive *Institutional* Conditions: |  |  |
| Bureaucratic Integrity | .70*** (74) |  |
| Law and Order | .56*** (74) |  |
| Civil Supremacy | .61*** (71) |  |
| Administrative Accountability | .70*** (73) |  |
| Institutional Functioning Index | .66*** (83) |  |
| Enduring Democracy | .61*** (89) |  |
| Civic Entitlements Index | .78*** (86) |  |
| Permissive *Cultural* Conditions: |  |  |
| Tightness-vs.-Looseness | .42* (18) |  |
| Individualism-vs.-Collectivism | .36^†^ (22) |  |
| Consanguine Marriages | - .62*** (28) |  |
| Patrilocal Household Formation | - .63*** (51) |  |
| Secular Values | .72*** (91) |  |
| Physical Security: |  |  |
| Internal Peace | .62*** (87) |  |
| External Peace | .34*** (74) |  |
| Encompassing Peace | .49*** (81) |  |
| External Linkages: |  |  |
| Percent Immigrants | .36*** (81) |  |
| Economic Exchange | .65*** (83) |  |
| Social Exchange | .70*** (85) |  |
| Political Exchange | .25** (87) |  |
| Global Exchange | .72*** (84) |  |
| Cultural Diffusion | .76*** (93) |  |
| MEAN (SD)^a)^ | .61 (.15) |  |
| *Notes*: Entries are bivariate Pearson correlations (*r*) with number of societies (*N*) in parentheses. Significance levels: * *p* < .100; ** p < .050; *** p < .005; ^†^ not significant (*p* > .100). Latest survey is for 5 societies from WVS round 5 with modal survey year 1996, for 34 societies from WVS round 4 with modal survey year 2000 and for 54 societies from round 5 with modal survey year 2006. | | |

# OA 12: Dynamic Societal-level Correlations with Emancipative Values

## Appendix-Table 5. Longitudinal Correlates of Rising Emancipative Values in Reproductive Freedoms

| CORRELATES: | Δ (*T*_2_ - *T*_1_) Emancipative Values |  |
| --- | --- | --- |
| Δ (*T*_2_ - *T*_1_) Existential Opportunities (sq.) | .48*** (50) |  |
| Δ (*T*_2_ - *T*_1_) Civic Entitlements | - .13^†^ (48) |  |
| Δ (*T*_2_ - *T*_1_) Secular Values | .62*** (49) |  |
| Δ (*T*_2_ - *T*_1_) Global Exchange | - .29* (37) |  |
| Δ (*T*_2_ - *T*_1_) Cultural Diffusion | .44*** (52) |  |
| *Notes*: Entries are bivariate Pearson correlations (*r*) with number of societies (*N*) in parentheses. Significance levels: * *p* < .100; ** p < .050; *** p < .005; ^†^ not significant (*p* > .100).  *T*_2_: Time of latest survey if at least ten years after first survey (15 surveys from WVS round 4 with modal year 2000 and 37 surveys from round 5 with modal survey year 2006; mean year of *T*_2_ is 2004)  *T*_1_: Time of earliest survey if at least ten years before last survey (23 surveys from WVS round 1 with modal survey year 1982, 22 surveys from round 2 with modal survey year 1990 and 7 surveys from round 3 with modal survey year 1996; mean year of *T*_1_ is 1987).  Δ (*T*_2_ - *T*_1_): Minimum time distance is 10 years, maximum is 27 years, mean time distance is 17 years. | | |

# OA 13: Proxy for Life Opportunities in the Cohort Analysis

Because measures for the life opportunities index are unavailable for decades before the 1960s, we rely on a proxy using data that Vanhanen (2003) had compiled to cover various decades back in time. Based on evidence that life opportunities depend on and strongly correlate with the size of the literate urban workforce (de Vries 1984; Bairoch 1995; Acemoglu, Johnson & Robinson 2001; Maddison 2007), we weight a society’s urbanization rate by its literacy rate using multiplication, after having standardized both variables into a range from minimum 0 to maximum 1.0. Thus, if the urbanization rate is 0.60 (60%) and the literacy rate is 0.50 (50%), the final score for the proxy of life opportunities is (0.50 * 0.60 =) 0.30.

That this measure is a reasonable proxy for life opportunities is evident from the fact that the proxy measure for 2000 correlates with our measure of life opportunities at *r* = .91 (*N* = 180; *p* < .001, two-tailed).

Data for this proxy of life opportunities are available from 1850 onward in decade-wise measures, for most nationally independent societies in each decade. For the longitudinal analysis in Table 3 of the article, we use the decade measures for 1940-50, 1950-60, 1960-70, 1970-80, 1980-90, 1990-2000. We are not going farther back in time than the decade 1940-50 because this is the farthest point back in time for which reasonable estimates of a society’s emancipative values can be calculated (see OA 15 below). Since emancipative values are one of the key three variables in Table 3, this restricts the possible temporal scope.

# OA 14: Proxy for Civic Entitlements in the Cohort Analysis

As a proxy for civic entitlements, we use the index of democratization by Vanhanen (2003), standardized into a 0-to-1 scale format (0 indicating no democracy, 1 indicating maximum democracy). The index is based on Dahl’s (1973) definition of democracy. Dahl defines democracy as the interaction between (a) political inclusion/participation and (b) political competition/pluralism. Political inclusion/participation is measured as the turnout in national parliamentary elections (calculated for the adult residential population); political competition/pluralism is the seat share not captured by the largest party in parliament. After standardization, these two indices are multiplied to yield the overall index of democratization. Note that this index has the intended property that, when the participation is 100% because all voters vote while pluralism is zero because all votes go to one party (a situation closely approximated in societies of the former Soviet bloc), the index of democratization yields a score of 0. The multiplicative combination treats the two components of participation and pluralism as necessary-yet-insufficient conditions of democracy—as it should be due to the theoretical notion of democracy.

Arguably, a high degree of both participation and pluralism requires a strong institutionalization of civic entitlements. Hence, the index of democratization is a reasonable proxy for civic entitlements for times for which a more direct measure of the latter is not available. Empirically, this is obvious from the fact that our measure of civic entitlements correlates with Vanhanen’s index of democratization in 2000 at *r* = .88 (*N* = 170; *p* < .001, two-tailed).

Data for this proxy of civic entitlements are available from 1850 onward in decade-wise measures. For the longitudinal analysis in Table 3 of the article, we use the decade measures for 1940-50, 1950-60, 1960-70, 1970-80, 1980-90, 1990-2000. We am not going farther back in time than the decade 1940-50 because this is the farthest point for which reasonable estimates of a society’s emancipative values can be calculated (see OA 15 below). Since emancipative values are one of the key variables in Table 3, this restricts the temporal scope of the analyses.

Another widely used indicator of democracy whose temporal coverage goes as far back as Vanhanen’s index of democratization is the ‘democracy-autocracy index’ from the Polity IV Project (data and description available at: www.systemicpeace. org/polity/polity4.htm). Using this index instead of that by Vanhanen as a proxy for civic entitlements in the analyses of Table 3, we obtain weaker results: civic entitlements are significantly but less strongly determined by emancipative values and continue to have no effect of their own on either emancipative values or life opportunities (these results are available upon request from the authors). From the viewpoint of nomological validity, this finding validates the democratization index by Vanhanen as a better measure of civic entitlements than the Polity IV autocracy-democracy index. This is not surprising if one takes into account that the Vanhanen index is entirely based on official statistical data while the Polity index is the result of subjective expert judgments (see Munck & Verkuilen 2002).

# OA 15: Emancipative Values in the Cohort Analysis

The cohort-related analysis is informed by the life-course theory of formative socialization. This theory assumes that the values of most people are formed by conditions prevalent during their upbringing (Inglehart 2008). Hence, we use proxy measures of life opportunities and civic entitlements, as they prevailed in a society during a given decade, to explain the emancipative values of those people in that society who grew up during the respective decade. This analysis, accordingly, is based on the following attribution scheme:

## Appendix-Table 6. Decennial Attribution Scheme

| Decade in which proxies for life opportunities (OA 13) and civic entitlements (OA 14) are measured | Birth year interval of country-cohort whose emancipative values today are predicted by the left-hand column |  |  |
| --- | --- | --- | --- |
| 1990-2000 | Cohort born after 1980 |  |  |
| 1980-1990 | Cohort born in 1970-1980 |  |  |
| 1970-1980 | Cohort born in 1960-1970 |  |  |
| 1960-1970 | Cohort born in 1950-1960 |  |  |
| 1950-1960 | Cohort born in 1940-1950 |  |  |
| 1940-1950 | Cohort born in 1930-1940 |  |  |

Based on this scheme, we have aggregate emancipative values for 85 countries divided into six cohorts (of all 95 countries surveyed once by the WVS, we lack cohort data for ten). This provides a data matrix of 510 country-cohort observations. Note, however, that there are empty cells because for decades in which a given country has not been independent, no proxy measure for life opportunities and civic entitlements is available: all three measures are available for 398 of the 510 country-cohorts. Based on multiple imputations (OA 16) to replace the missing values with expected values, we replicated the cohort-related analyses reported in the article with the imputed full data matrix. Results remained the same.

# OA 16: Results from Multiple Imputations and ‘SUR’

Based on the matrix consisting of 85 societies and six decades each we ran a standard, multiple imputation algorithm in SPSS to replace missing values with expected values. For each missing value, five different estimates are produced based on confidence intervals, yielding five different data matrices. Each of these data matrices is complete, including (85 * 6 =) 510 country-per-cohort observations. We reran with each of these five datasets the same regressions as those reported in the article, using panel-corrected standard errors. Results remained the same and can be obtained upon request from the authors.

# OA 17: Temporally Ordered Panel Regressions with Country-Cohorts

|  | DEPENDENT VARIABLE:  Emancipative Values in Reproductive Freedoms (2000-2008) | |
| --- | --- | --- |
| PREDICTORS (during country-cohorts’ upbringing): | Model 1 | Model 2 |
| Constant | .13 (11.68) *** | .06 ( 4.86) *** |
| Life Opportunities | .44 (14.12) *** | .21 ( 6.47) *** |
| Civic Entitlements | .08 ( 4.16) *** | .09 ( 3.27) *** |
| Secular Values | .11 ( 3.36) *** | .52 (11.23) *** |
| East Asia (dummy) |  | - .20 (-6.81) *** |
| Adj. R^2^ | .69 | .78 |
| *N* (country-cohorts) | 306 | 301 |
| *Note*: Regressions calculated with panel-corrected standard errors in STATA. Entries are unstandardized regression coefficients with their T-values in parentheses. Test statistics of heteroskedasticity (White-test) and multicollinearity (variance inflation factors) reveal no violation of OLS assumptions. Significance levels: * *p* < .100; ** p < .050; *** p < .005; ^†^ not significant (*p* > .100). | | |
| PREDICTORS (during country-cohorts’ teenage years): | DEPENDENT VARIABLE:  Secular Values (2000-2008) | |
| Constant | .16 (12.63) *** |  |
| Life Opportunities | .46 (13.56) *** |  |
| Civic Entitlements | .03 ( 0.86) ^†^ |  |
| East Asia (dummy) | .36 (11.95) *** |  |
| Adj. R^2^ | .66 |  |
| *N* (country-cohorts) | 301 |  |
| *Note*: Regressions calculated with panel-corrected standard errors in STATA. Entries are unstandardized regression coefficients with their T-values in parentheses. Test statistics of heteroskedasticity (White-test) and multicollinearity (variance inflation factors) reveal no violation of OLS assumptions. Significance levels: * *p* < .100; ** p < .050; *** p < .005; ^†^ not significant (*p* > .100). | | |

Note that one obtains similar results when using a two-stage least squares regression in which secular values are in the first stage regressed on life opportunities, civic entitlements and the East Asia dummy, and then the instrumented values in secular values are used to predict emancipative values in the second stage.

# OA 18: Additional Individual-level Data for the Multi-level Models

*Female Sex*: V235 in the wave-5 questionnaire advices interviewers to code the sex of the respondent by observation. We recoded the variable into 0 for male and 1 for female.^[[4]](#footnote-4)^

*Formal Education*: The variable is measured in nine ascending categories, indicating the highest achieved level of education from 1 “no formal education” to 9 “university degree” based V238 of the WVS round-five questionnaire. We recode the scores into a range from minimum 0 to maximum 1.0, with fractions for intermediate positions.^[[5]](#footnote-5)^

*Birth Year (indexed)*: Information on the respondents’ birth years [V236 in the wave-5 questionnaire of the WVS] has been indexed, setting 1900 at 0, 1990 at 1.0, and years in between at their corresponding intermediate position between 1900 and 1990. The formula for this calculation: birth year minus 1900 divided by 1990 minus 1900.

# OA 19: Temporal Distances per Society for the Change Measures

Temporal distances from earliest to latest available surveys are as follows (rounded): Argentina (round I to V): 25 years; Australia (III to V): 10 years; Austria (II to IV): 10 years; Belgium (I to IV): 20 years; Brazil (II to V): 15 years; Bulgaria (II to V): 10 years; Belarus (II to IV): 10 years; Canada (I to V): 25 years; Chile (II to V): 15 years; China (II to V): 15 years; Taiwan (III to V): 10 years; Colombia (III to V): 10 years; Czech R. (II to IV): 10 years; Denmark (I to IV): 20 years; Estonia (II to IV): 10 years; Finland (II to V): 15 years; France (I to V): 25 years; Hungary (I to IV): 20 years; Iceland (I to IV): 20 years; India (II to V): 15 years; Ireland (I to IV): 20 years; Italy (I to V): 25 years; Japan (I to V): 25 years; S. Korea (I to V): 25 years; Latvia (II to IV): 10 years; Lithuania (II to IV): 10 years; Malta (I to IV): 20 years; Mexico (II to V): 15 years; Moldova (III to V): 10 years; Netherlands (I to V): 25 years; New Zealand (III to V): 10 years; Nigeria (II to IV): 10 years; Norway (I to V): 25 years; Poland (II to V): 15 years; Portugal (II to IV): 10 years; Romania (II to V): 15 years; Russia (II to V): 15 years; Slovakia (II to IV): 10 years; Slovenia (II to V): 15 years; S. Africa (II to V): 15 years; Spain (I to V): 25 years; Sweden (I to V): 25 years; Switzerland (II to V): 15 years; Turkey (II to V): 15 years; Ukraine (III to V): 10 years; UK (I to V): 25 years; USA (I to V): 25 years; Uruguay (III to V): 10 years; Germany (W.) (I to V): 25 years; Germany (E.) (II to V): 15 years; Yugoslavia (III to V): 10 years.

Note that, in order to compare univariate change measures in emancipative values, one needed to standardize the differences in covered time spans (for instance, by calculating the mean *annual* change for each society). However, in a bivariate analyses in which change in the predictor variables of emancipative values is measured for each society over the same time span as change in emancipative values, the differences in time spans are not a source of inequivalence between emancipative values and its predictors: if the time span of change in emancipative values is short, the time span of change in the predictors is equally short; if the time span of change in emancipative values is long, the time span of change in the predictors is equally long.

## Appendix-Table 9. Variability of Key Variables in the Longitudinal Sample: Standard Deviation (Range)

|  | Emancipative Values | Secular Values | Life Opportunities (sq.) | Civic Entitlements |
| --- | --- | --- | --- | --- |
| Longitudinal Sample (*N* = 52) | .16 (.71) | .17 (.70) | .24 (.99) | .29 (1.0) |
| Other Societies (*N* = 41) | .14 (.75) | .16 (.65) | .19 (.74) | .26 (.99) |

# OA 20: Descriptive Statistics for the Cross-Sectional and Dynamic Analyses

| **Descriptive Statistics (time-pooled cross section)** | | | | | |
| --- | --- | --- | --- | --- | --- |
|  | N | Minimum | Maximum | Mean | Std. Deviation |
| ctrnum Ctr ID number | 252 | 2.00 | 203.00 | 100.5000 | 57.18771 |
| wave Wave | 252 | 1 | 5 | 3.38 | 1.274 |
| ctrwave Ctr ID Wave | 252 | 23 | 2035 | 1008.58 | 571.863 |
| s024a Country - wave (EVS separate) | 252 | 83 | 9124 | 4748.42 | 2747.503 |
| DelDum delta dummy (1 yes, longterm change measure) | 252 | .00 | 1.00 | .7460 | .43615 |
| CulZon 10 culture zones | 252 | 1.00 | 10.00 | 5.0556 | 2.88506 |
| earliest earliest surv even if only surv | 97 | 1.00 | 1.00 | 1.0000 | .00000 |
| latest latest surv even if only surv | 97 | 1.00 | 1.00 | 1.0000 | .00000 |
| earliestrep earliest surv if at least 2 survs | 69 | 1.00 | 1.00 | 1.0000 | .00000 |
| latestrep latest surv if at least 2 survs | 69 | 1.00 | 1.00 | 1.0000 | .00000 |
| EmaVal Emancipative Values: repro free at Time of Survey | 241 | .03 | .80 | .3457 | .14791 |
| lgEmaVal Emancipative Values at earliest survey | 52 | .08 | .60 | .3150 | .09924 |
| dEmaVal change Emanc Values earliest to latest survey | 52 | -.16 | .33 | .1049 | .10761 |
| SecVal Secular Values: disbelief at time of survey | 243 | .05 | .94 | .4145 | .18106 |
| SecVal_e Secular Values at earliest survey | 50 | .10 | .94 | .4496 | .17246 |
| SecVal_l Secular Values at latest survey | 53 | .05 | .75 | .4620 | .16484 |
| dSecVal change Secular Values earliest to latest survey | 50 | -.19 | .23 | .0131 | .09811 |
| LifOpp Life Opportunities (f_LifOpp standardized 0-1) | 241 | .00 | 1.00 | .6248 | .19950 |
| sqLifOpp LifOpp squared | 241 | .00 | 1.00 | .4300 | .22769 |
| LifOpp_e LifOpp at earliest survey | 53 | .00 | .89 | .6012 | .16190 |
| sqLifOpp_e squared LifOpp_e | 53 | .00 | .79 | .3889 | .16468 |
| LifOpp_l LifOpp at latest survey | 53 | .08 | 1.00 | .7256 | .18330 |
| sqLifOpp_l squared LifOpp_l | 53 | .01 | 1.00 | .5605 | .23847 |
| dLifOpp Change in LifOpp (LifOpp_l minus LifOpp_e) | 53 | -.01 | .35 | .1244 | .08532 |
| dsqLifOpp Change in squared LifOpp | 53 | -.02 | .50 | .1716 | .13434 |
| GloExc Global Exchange at time of survey | 222 | .25 | .93 | .6288 | .15479 |
| dGloExc change Global Exchange earliest to latest survey | 74 | .00 | .38 | .1882 | .07503 |
| DifEmaVal CultZone Diffus EmaVal | 240 | .11 | .65 | .3745 | .15001 |
| DifForSym CultZone Diffus ForSym | 234 | .59 | .94 | .8284 | .09833 |
| DifdEmaVal CultZone Difus dEmaVal | 52 | -.12 | .69 | .4518 | .15546 |
| DifdForSym CultZome Difus dForSym | 51 | -.16 | .09 | .0000 | .04736 |
| Valid N (listwise) | 0 |  |  |  |  |

| **Descriptive Statistics (cross-section, latest survey)** | | | | | |
| --- | --- | --- | --- | --- | --- |
|  | N | Minimum | Maximum | Mean | Std. Deviation |
| ctrnum Ctr ID number | 97 | 2.00 | 203.00 | 99.4948 | 59.57228 |
| wave Wave | 97 | 3 | 5 | 4.55 | .595 |
| ctrwave Ctr ID Wave | 97 | 24 | 2035 | 999.60 | 595.798 |
| s024a Country - wave (EVS separate) | 97 | 84 | 9124 | 4609.08 | 2781.866 |
| DelDum delta dummy (1 yes, longterm change measure) | 97 | .00 | 1.00 | .5361 | .50129 |
| CulZon 10 culture zones | 97 | 1.00 | 10.00 | 5.6186 | 2.88113 |
| earliest earliest surv even if only surv | 28 | 1.00 | 1.00 | 1.0000 | .00000 |
| latest latest surv even if only surv | 97 | 1.00 | 1.00 | 1.0000 | .00000 |
| earliestrep earliest surv if at least 2 survs | 0 |  |  |  |  |
| latestrep latest surv if at least 2 survs | 69 | 1.00 | 1.00 | 1.0000 | .00000 |
| EmaVal Emancipative Values: repro free at Time of Survey | 93 | .03 | .80 | .3414 | .17534 |
| lgEmaVal Emancipative Values at earliest survey | 52 | .08 | .60 | .3150 | .09924 |
| dEmaVal change Emanc Values earliest to latest survey | 52 | -.16 | .33 | .1049 | .10761 |
| SecVal Secular Values: disbelief at time of survey | 95 | .05 | .75 | .3770 | .18683 |
| SecVal_e Secular Values at earliest survey | 50 | .10 | .94 | .4496 | .17246 |
| SecVal_l Secular Values at latest survey | 53 | .05 | .75 | .4620 | .16484 |
| dSecVal change Secular Values earliest to latest survey | 50 | -.19 | .23 | .0131 | .09811 |
| LifOpp Existential Opportunities (f_LifOpp standardized 0-1) | 91 | .05 | 1.00 | .6111 | .23412 |
| sqLifOpp LifOpp squared | 91 | .00 | 1.00 | .4276 | .26371 |
| LifOpp_e LifOpp at earliest survey | 53 | .00 | .89 | .6012 | .16190 |
| sqLifOpp_e squared LifOpp_e | 53 | .00 | .79 | .3889 | .16468 |
| LifOpp_l LifOpp at latest survey | 53 | .08 | 1.00 | .7256 | .18330 |
| sqLifOpp_l squared LifOpp_l | 53 | .01 | 1.00 | .5605 | .23847 |
| dLifOpp Change in LifOpp (LifOpp_l minus LifOpp_e) | 53 | -.01 | .35 | .1244 | .08532 |
| dsqLifOpp Change in squared LifOpp | 53 | -.02 | .50 | .1716 | .13434 |
| GloExc Global Exchange at time of survey | 87 | .34 | .93 | .6609 | .15525 |
| dGloExc change Global Exchange earliest to latest survey | 39 | .00 | .38 | .1931 | .08103 |
| DifEmaVal CultZone Diffus EmaVal | 93 | .14 | .63 | .3408 | .14344 |
| DifForSym CultZone Diffus ForSym | 91 | .60 | .93 | .8155 | .09784 |
| DifdEmaVal CultZone Difus dEmaVal | 52 | -.12 | .69 | .4518 | .15546 |
| DifdForSym CultZome Difus dForSym | 51 | -.16 | .09 | .0000 | .04736 |
| Valid N (listwise) | 0 |  |  |  |  |

# OA 21: Descriptive Statistics for the Country-Cohort Analyses

| **Descriptive Statistics (time-pooled cross sectional)** | | | | | |
| --- | --- | --- | --- | --- | --- |
|  | N | Minimum | Maximum | Mean | Std. Deviation |
| ctrnum ctr id number | 510 | 2.00 | 201.00 | 99.2706 | 56.56730 |
| cohort birth cohort number | 510 | 3.00 | 8.00 | 5.5000 | 1.70950 |
| ctrcoh Ctr Cohort | 510 | 23.00 | 2018.00 | 998.2059 | 565.67562 |
| LifOpp Life Opportunities in given D | 398 | .00 | .97 | .4980 | .31861 |
| EmaVal Emanc Values (choice comp) in given D | 508 | .02 | .80 | .3113 | .17635 |
| CivEnt Civic Entitlements in given D | 423 | .00 | 1.00 | .2966 | .29634 |
| Lg1LifOpp 1 D Lagged Life Opportunities | 314 | .00 | .97 | .4576 | .31486 |
| Lg1EmaVal 1 D Lagged Emanc Vals | 423 | .02 | .78 | .3003 | .17146 |
| Lg1CivEnt 1 D Lagged Civic Entitlements | 340 | .00 | 1.00 | .2663 | .29314 |
| Lg2LifOpp 2 D Lagged Life Opportunities | 239 | .00 | .96 | .4247 | .30746 |
| Lg2EmaVal 2 D Lagged Emanc Vals | 338 | .02 | .78 | .2884 | .16560 |
| Lg2CivEnt 2 D Lagged Civic Entitlements | 257 | .00 | .89 | .2309 | .27989 |
| Valid N (listwise) | 230 |  |  |  |  |

# OA 22: Descriptive Statistics for the Multi-level Analyses

| **Descriptive Statistics (individual-level data)** | | | | | |
| --- | --- | --- | --- | --- | --- |
|  | N | Minimum | Maximum | Mean | Std. Deviation |
| ctrnum country id number | 138431 | 2.00 | 203.00 | 98.7227 | 58.20650 |
| casenum respondent id | 138431 | 7.00 | 347922.00 | 171591.2661 | 101136.92635 |
| EmaVal | 138431 | .00 | 1.00 | .2926 | .27827 |
| EVrest | 138431 | .00 | 1.00 | .4454 | .18398 |
| SecVal | 138431 | -.01 | 1.00 | .3470 | .30760 |
| FemSex | 138431 | .00 | 1.00 | .5124 | .49985 |
| BirYea | 138431 | .03 | 1.00 | .6962 | .17604 |
| ForEdu | 138431 | .00 | 1.00 | .4834 | .33171 |
| SamWeig | 138431 | .02 | 22.46 | 1.1157 | .56917 |
| Valid N (listwise) | 138431 |  |  |  |  |

| **Descriptive Statistics (societal-level data)** | | | | | |
| --- | --- | --- | --- | --- | --- |
|  | N | Minimum | Maximum | Mean | Std. Deviation |
| ctrnum Ctr ID number | 90 | 2.00 | 201.00 | 98.1667 | 58.54879 |
| CulZon 10 culture zones | 90 | 1.00 | 10.00 | 5.6889 | 2.91314 |
| ConDum East Asia Dummy | 90 | .00 | 1.00 | .0667 | .25084 |
| EValSL emanc vals soc level | 86 | .03 | .80 | .3346 | .17470 |
| SValSL secul vals soc level | 90 | .05 | .75 | .3664 | .18410 |
| CivEnt civic entitlements | 90 | .00 | 1.00 | .4939 | .32854 |
| sqLOpp squared exist opport | 90 | .00 | 1.00 | .4276 | .26727 |
| Valid N (listwise) | 86 |  |  |  |  |

# OA 23: Response Section

This section clarifies issues that have been raised in the review process and for which there was not enough space to incorporate them into the article itself, without exceeding the word count limit.

## REVIEWER #1

The first reviewer summarizes our article’s achievements with the following comment: “The authors have handled a huge volume of data and analyzed them painstakingly. The paper also poses an interesting question. However, the narrow focus on material conditions (that too at the aggregate level) weakens the conceptual strength of the paper.”

**Author Response:**

We are pleased about the positive aspects of this evaluation and wish to address first the reviewer’s general criticism about our too narrow focus on material conditions and the focus of most of the analyses on the aggregate level. After that, we proceed in addressing the fifteen (very useful) specific points that this reviewer raises.

On p. 12 of the revised manuscript, we now point out that the “objective” existential conditions we are examining include material conditions but only as one component among many others, which reach from education to health to fertility. We hope it is clearer now from the revised manuscript that, if anything can be said about the scope of the living conditions covered, it is the very breadth of this scope.

Nevertheless, we condense the broad scope of living conditions into a single compact “index of life opportunities.” This is justified on the basis of both a factor analysis and a reliability analysis of the included components, which largely merge into a latent overall dimension of restrictive versus permissive existential conditions. Summarizing these components in an overall index is to cover their shared variance. This summary creates a more reliable measure that averages out each single component’s specific measurement error: where a single component goes out of line of the other components, the deviation is evened out through the aggregation of component scores. Hence, our index of existential opportunities outperforms the widely known “human development index” (HDI) in predicting emancipative values and change therein. One reason is that our index of life opportunities is broader in terms of covered domains. We footnote this clarification on p. 12 of the revised manuscript.

The final step of the analyses uses a multilevel model to examine emancipative values in the domain of reproductive freedoms at the *individual* level (see Table 3 and Figure 7). The revised manuscript points this out more clearly now at pp. 7-8 (points 5 and 6) and p. 17. Hence, our analyses do move *beyond* the aggregate level. Still, we retain a focus on aggregate patterns because our whole theory is about socio-cultural *trends*. Trends are an aggregate phenomenon by definition and need to be looked at as such.

Our subsequent responses follow the numbers that the reviewer has pointed out.

(1) Abstract: Traditional Reproduction Norms

**Reviewer Suggestion:**

The reviewer wants us to tone down our statement in the abstract that traditional family, fertility and sex norms are most resistant to change. We should say that this is just one of the fields where resistance is strong.

**Author Response:**

We are grateful for this advice and re-wrote the abstract accordingly. What we now say is that family, fertility and sex norms belong to those domains where tradition has *some* of its strongest stance.

(2) Introduction: Focus on Reproductive freedoms

**Reviewer Suggestion:**

The paper’s focus on reproductive freedoms should be stated more clearly in the introduction.

**Author Response:**

In the abstract and in the introduction at pp. 1-2, we now point out more clearly that our analyses focus on emancipative values *specifically* in the domain of reproductive freedoms.

(3) Literature Review

**Reviewer Suggestion:**

The reviewer suggests to shorten the literature review.

**Author Response:**

We have considerably shortened the literature review and limited ourselves to a condensed reference to the relevant literature on pp. 4-5.

(4) Doubtful Assumption that Prosperity brings Tolerance

**Reviewer Suggestion:**

The reviewer raises doubts about the hypotheses that economic prosperity brings more tolerance because s/he believes that the US has not become more tolerant of African Americans in the wake of rising prosperity.

**Author Response:**

This is an important comment. Our whole theoretical framing on pp. 4-7 of the revised manuscript is now written in a way that deliberately avoids a narrow focus on material conditions and instead emphasizes rising life opportunities in a much broader sense. Indeed, we focus on the broad expansion of life horizons and opportunities for self-development emerging with rising life expectancies, widening access to education and diminishing fertility pressures. We argue that this broad ascension of life opportunities makes people more supportive of universal freedoms, including those related to reproduction. Improving material conditions are a significant contributor to the ascension of life opportunities but they are only part of the story.

We also outline on pp. 5-6 and 12 of the revised manuscript that our notion of life opportunities fits the “life history” approach in evolutionary psychology. This approach juxtaposes “short life histories,” which are characterized by high mortality, fertility, inequality and poverty and few opportunities for education, and “long life histories,” which are characterized by the opposite: low mortality, fertility, inequality and poverty with abundant opportunities for education. Woodley (2011), for instance, summarizes these indicators in a “life history factor,” much the same way in which we summarize our indicators into the “life opportunities index.”

(5) Shorter Description of Mechanism

**Reviewer Suggestion:**

The reviewer wants us to shorten the review and get more directly to a clearer description of the mechanism of how ascending life opportunities are conducive to emancipative values in the domain of reproductive freedoms. S/he also wants us to relate the mechanism to Abraham Maslow’s hierarchy of human motivations.

**Author Response:**

As mentioned before, we have indeed shortened the literature review considerably. And we tried to explain in clearer terms than we did before (see pp. 5-7) how the two key premises of Welzel’s (2013; 2014) evolutionary emancipation theory inform the hypothesis that rising opportunities for self-realization on a mass scale strengthen emancipative values, which eventually include an emphasis on reproductive freedoms.

On p. 7 (footnote 1) of the revised manuscript, we point out that the utility of ladder of freedoms captures Maslow’s (1954) hierarchy of motivations: higher rungs on the utility ladder correspond with higher-ordered motivations. Specifically, the more evolved drive for self-development and learning lends itself more closely to freedoms than the more basic drive for defence and survival (Lawrence & Nohria 2002). Yet, the emphasis of the utility ladder on shared utilities gives the utility ladder a social dimension that the hierarchy of motivations lacks. For this reason, the utility ladder is a more adequate tool to describe social change than the hierarchy of motivations.

(6) Intra-Societal Differences

**Reviewer Suggestion:**

The reviewer criticizes that our approach ignores intra-societal differences.

**Author Response:**

In response to this critique, we wish to point out that we are fully aware of *intra*-societal differences but have chosen to focus more on *inter*-societal differences because we are interested in cross-national social trends, in which case national populations naturally become the prime unit of analysis. We believe that this is justified by the fact that the nation is still the most powerful unit in shaping collectives, both economically and culturally, and much more so than social class or religion. For instance, Firebaugh (2012) shows that 70 per cent of the global income inequality is due to differences between countries while only 30 per cent is due to differences within countries (be it by race, class, religion or gender). Similarly, Inglehart and Welzel (2005; 2010) demonstrate with broad empirical evidence that cultural differences between nations dwarf those within nations, even considering different income groups or religious denominations. We place a footnote with a link to this clarification at p. 15 of the revised manuscript.

Nevertheless, we wish to emphasize that we do give intra-societal differences some consideration. The multilevel analyses in Table 3 and Figure 7 explicitly examine individual-level differences in emancipative values within societies. These analyses are conducted to test our assumptions number 5 and 6 (pp. 7-8), namely that even individual-level values are shaped more strongly by societal-level conditions than by individual-level characteristics. The results fully confirm this expectation.

(7) Median Instead of Mean

**Reviewer Suggestion:**

The reviewer suspects that our mean scores in population value measures can be biased by outliers and that using the median would be a better approach.

**Author Response:**

We have checked this issue painstakingly. With an average of some 1,000 respondents per country, single outliers hardly affect the location of the mean. Also, median and mean are very close to each other in every of our national samples. What is more, when there is a normal distribution with most respondents clustered around the country mean in each sample, the mean is the more appropriate figure to represent the central tendency, which is what we are interested in. For all these reasons, we stick to our choice. We footnote this clarification at p. 14 of the revised manuscript.

(8) Endogeneity

**Reviewer Suggestion:**

The reviewer remarks that our hypotheses have an inbuilt endogeneity because emancipative values in the domain of reproductive freedoms are part of what defines permissive existential conditions.

**Author Response:**

In a sense, we agree with this view and footnote a link to this clarification at p. 20 of the revised manuscript. Indeed, we believe that “permissiveness” provides a conceptual bridge between opportunity endowments and emancipative values, which is actually the reason why we assume the two to be empirically linked. However, we still see a conceptual distinction between basic existential conditions, which relate to life circumstances in an “objective” sense, and their psychological manifestation in “subjective” values. Our existential conditions indicators address purely objective phenomena (things existing beyond people’s perception) while our measure of emancipative values is purely subjective (telling us what people wish to prioritize). Hence, despite the supposed link between the two, they are conceptually speaking not the same, for which reason their empirical link becomes a worthwhile object of research. Indeed, the strength of this link tells us to what extent permissive configurations cut through the “objective/subjective” boundary, or to what extent subjective values adjust to objective utilities.

Apart from this theoretical argument, we addressed the issue of endogeneity empirically. Specifically, we used an instrumental variables approach and tested if life opportunities retain a significant and positive effect on emancipative values when we instrument them with fertility and mortality data from a hundred years ago, that is, in 1900. These data are definitely exogenous to emancipative values today. Hence, if we use these exogenous data as instruments for life opportunities today, we “de-endogenize” life opportunities from emancipative values and isolate their truly exogenous impact on these values.

Indeed, if we “instrument” scores on the index of contemporary life opportunities with the fertility and mortality data from 1900, the instrumented scores retain a significant and strongly positive effect on emancipative values. This is an important finding: the instrumented life opportunity scores are definitely exogenous to emancipative values because they are derived from original opportunity endowments dating back a hundred years. Accordingly, the determination of emancipative values by life opportunities does not reflect the latter one’s endogeneity to emancipative values.

We conducted the same instrumental variables test with secular values and the result was the same: isolating the exogenous part of these values, their effect on emancipative values retains its positivity and significance. Detailed results are available upon request by the authors.

Further (successful) treatments of the endogeneity problem are reported in the dynamic model section on p. 21 and the temporal order model section on p. 25 of the revised manuscript.

(9) Assymetries in Gains in Emancipative Values within Same Groups

**Reviewer Suggestion:**

The reviewer criticizes that we “fail to consider asymmetries in gains in ERF-Values within same social groups.”

**Author Response:**

This point is intimately connected to the next one, so we respectfully refer to our response to point 10.

(10) Homogenous Societies

**Reviewer Suggestion:**

The reviewer suspects that using country averages involves an implicit assumption of homogenous societies.

**Author Response:**

In response to this important objection, we wish to point out that our usage of country averages does not assume intra-societal homogeneity. Rather, it involves three more modest but demonstrably valid assumptions: (1) despite intra-societal differences (which we do not deny), nations show distinct mass tendencies in given variables; (2) the differences between these mass tendencies are very significant, considerable in size and at times outmatch intra-societal differences; (3) country averages provide valid representations of each nation’s mass tendency in a given characteristic.

As concerns our main database, the World Values Surveys, these points have been demonstrated with abundant evidence by Inglehart and Welzel (2005; 2010) and Minkov and Bond (2013). We footnote a link to this clarification on p. 15 of the revised manuscript.

(11) Factor Scores

**Reviewer Suggestion:**

The reviewer asks why we do not use factor analyses to create index scores on emancipative values in reproductive freedoms.

**Author Response:**

On p. 10 of the revised manuscript, we explicitly mention the existence of such a factor analysis and navigate readers to its location: Appendix-Table 1 in OA 1 (p. 6). This analysis shows equally strong loadings for all three components of our measure of emancipative values in reproductive freedoms. Hence, using factor scores would not make any difference. Moreover, for replication purposes, we use the index construction rules outlined and justified on the basis of extensive reliability and validity checks by Welzel (2013).

(12) Shortening

**Reviewer Suggestion:**

The reviewer suggests to cut the paper shorter.

**Author Response:**

We shortened the paper by 1,100 words, bringing it down to some 10,000 words.

(13) Several Papers

**Reviewer Suggestion:**

The reviewer suggests to divide the paper into several papers, one for each hypothesis.

**Author Response:**

Since the hypotheses build on each other and are interlinked within a single general theory, we respectfully dis-consider this possibility.

(14) Length of Trend

**Reviewer Suggestion:**

The reviewer suggests that traditional norms should have given in already in the past under improving living conditions.

**Author Response:**

The cohort-related analyses on pp. 23-26 (Figures 5 and 6) are based on data that reach much farther back in time. The analyses actually show that the reviewer is accurate in this assumption.

(15) Other Norms

**Reviewer Suggestion:**

The reviewer asks if other norms also have been affected by improving living conditions.

**Author Response:**

The answer is a clear yes and p. 12 of the revised manuscript reports parallel value changes in three other domains linked to emancipative values. A footnote on p. 12 also mentions a publication showing with the same data that people’s willingness to fight for their countries in the case of war has been in decline in most countries and that this trend is most pronounced where life opportunities expanded for large population segments. Our article does not leave enough room to go deeper into these findings than mentioning them in passing. Also, we think it is legitimate to retain the article’s focus on a specific set of values.

## REVIEWER #3

The third reviewer notes: “This manuscript reports a study with a very large sample of people from many countries. It tests several interesting hypotheses that are theory driven. It is well written, the analyses seem to be appropriate, and they are explained clearly.”

We are grateful to such a positive evaluation and happy to conduct the many specific corrections the reviewer is asking for. These include the elimination of the mentioned typos and the dissolution of capital letter abbreviations upon their first appearance. Apart from these important but relatively small corrections, our revision addresses the following points raised by the third reviewer.

Recentness of Trend

**Reviewer Suggestion:**

The reviewer questions that emancipatory breakthroughs into traditional family, fertility and sex norms are so recent. S/he mentions, for instance, the liberalization of abortion laws in the U.S. in the aftermath of 1968.

**Author Response:**

In response to this important point, we wish to sharpen our argument a bit more. Our statement of recentness applies more specifically to the advancement of the same-sex marriage issue and the cultural clashes about the legalization versus criminalization of homosexuality. These divergent developments in law are linked to generational value changes that have been breaking up the consensual rejection of homosexuality in most of the world’s cultures. The shift among younger cohorts towards greater acceptance of homosexuality is visible—albeit to different degrees--throughout countries of all cultural zones of the globe. And these generational shifts are linked everywhere with similar shifts in the acceptance of divorce and abortion. Hence, support for the freedoms of sexual orientation, divorce and abortion merge into a tightly-knit compound. For these reasons, we opt against separating the three components.

Moreover, an early legalization of divorce and abortion is a specifically Western phenomenon and one that is limited to some Protestant Western societies, whereas in many Catholic countries (i.e., Ireland) divorce and abortion have been legalized rather late. Even in the vanguard countries of this movement, reproductive freedoms have usually been the most recent freedoms that have been legalized, often a long time after the legalization of political freedoms. We footnote a link to this clarification on p. 1.

Incomplete Web-site Reference to Appendix

**Reviewer Suggestion:**

The reviewer points out that the website address to the Online Appendix is incomplete and that s/he could not access the Online Appendix.

**Author Response:**

We regret this mistake and hope that, in this submission round, the appendix will be made available to the reviewers. This was our intention already at the first submission and we uploaded the Online Appendix as supplementary material in the manuscript submission system. We do not know why the Online Appendix has not been available to the reviewers and hope that this will be rectified with this round of submission.

As concerns the incomplete website reference to the Online Appendix, we could not yet provide a complete web address as we do not know it yet. The address will only been known once SIR accepts this article for publication. Despite the incomplete address at the current stage, the Online Appendix should be available to the reviewers for download via the online submission system.

Feminine Form: Have only Women Been Analyzed?

**Reviewer Suggestion:**

The reviewer remarks that the consistent use of the feminine form in personal pronouns is confusing because it might suggest that our analyses only covered women. For this reason, the reviewer recommends the gender-neutral form of pronouns. S/he also asks about the effect of gender on emancipative values.

**Author Response:**

We follow this advice and use pronouns in the gender-neutral form. This should make it sufficiently obvious that we did not limit the multilevel analyses to women but included both sexes in each sample. This should also be clear from the fact that the multilevel models in Table 3 include gender as a variable. As is obvious from these models, female sex has a very small but nevertheless consistent and significant effect on support of reproductive freedoms: being a women, adds on average .02 scale points to the constant term in emancipative values. Furthermore, the strength of the positive female effect varies, being more strongly positive in societies with predominant secular norms. We footnote this finding at p. 27 of the revised manuscript. Unfortunately, the given space limitation and focus of the paper do not leave enough room to elaborate this finding any further.

Deficient Conclusion

**Reviewer Suggestion:**

The reviewer advises us to point out more clearly what our findings add to the existing literature and in what respect they are novel. We are also advised to comment on the limitations of our findings.

**Author Response:**

We are grateful to this important advice and have re-written the conclusion accordingly, pointing out where our findings are new, where they are limited and what this means for the future research agenda.

Listing of Countries

**Reviewer Suggestion:**

The reviewer wants us to list in the text the societies belonging into Welzel’s cultural zones as well as the dozen postindustrial societies covered by the analyses in Figure 5.

**Author Response:**

The dozen postindustrial societies are listed in the footer of Figure 5. Welzel’s cultural zone scheme, by contrast, applies to more than ninety countries and is simply too long to incorporate it in Figure 5 or anywhere else in the manuscript. Thus, we amended the footer in Figure 5, mentioning that the culture zone classification can be found on p. 6 of the OA.

1. Assume the variable name for the original civil liberties rating by Freedom House in 1981 is “civlib81”, the SPSS-Syntax to create the inverted and standardized rating is: Compute cl81 = 1 – ((civlib81 – 1) / 6). Assume the variable name for the original political rights rating in 1981 is “polrig81”, the SPSS-Syntax to create the inverted and standardized rating is: Compute pr81 = 1 – ((polrig81 – 1) / 6). Then we calculate the summary Freedom House (FH) measure: Compute FH81 = (cl81 + pr81) / 2. [↑](#footnote-ref-1)
2. Assume the variable name for the original integrity rights rating by Cingranelli and Richards in 1981 is “intrig81”, the SPSS-Syntax to create the standardized rating is: Compute ir81 = intrig81 / 8. Assume the variable name for the original empowerment rights rating in 1981 is “empow81”, the SPSS-Syntax to create the standardized rating is: Compute er81 = empow81 / 14. Then we calculate the summary Cingranelli/Richards (CR) measure: Compute CR81 = (ir81 + er81) / 2. [↑](#footnote-ref-2)
3. Syntax: Compute CitRig81 = FH81 * CR81. [↑](#footnote-ref-3)
4. SPSS syntax: Recode v235 (2=1) (1=0) into female. Recode female (sysmiss=-99). Mis val female (-99). Val lab female 1"female" 0"male". Var lab female "female dummy". [↑](#footnote-ref-4)
5. SPSS syntax: compute educ = (v238 – 1) / (9 – 1). [↑](#footnote-ref-5)
